# 2D Interfacial Crystallization Stabilized by Short-Chain Aliphatic Interfaces

**DOI:** 10.1021/acs.langmuir.4c04718

**Published:** 2025-03-11

**Authors:** Hamish W. A. Swanson, Kenny Barriales, Emmet A. Sherman, Tai-De Li, Alan R. Kennedy, Tell Tuttle, Rein V. Ulijn, King Hang Aaron Lau

**Affiliations:** † Department of Pure and Applied Chemistry, University of Strathclyde, 295 Cathedral Street, Glasgow G1 1XL, U.K.; ‡ Nanoscience Initiative at Advanced Science Research Center, The Graduate Center, The City University of New York, 85 Saint Nicholas Terrace, New York, New York 10031, United States; § Department of Chemistry, Hunter College, The City University of New York, 695 Park Avenue, New York, New York 10065, United States; ∥ Ph.D. Program in Chemistry, The Graduate Center, The City University of New York, 365 Fifth Avenue, New York, New York 10016, United States; ⊥ Department of Physics, The City College of New York, The City University of New York, 160 Convent Avenue, New York, New York 10031, United States; # Advanced Science Research Center, The Graduate Center, The City University of New York, 85 Saint Nicholas Terrace, New York, New York 10031, United States

## Abstract

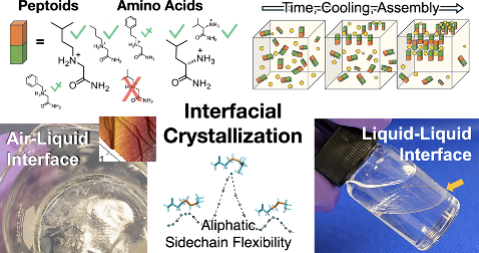

We report the discovery and in-depth investigation of interfacial crystallization (IFC), the assembly and formation of membrane-like crystalline sheets from both chiral amino acid and achiral *N*-substituted glycine “peptoid” amide monomers selectively at vapor–liquid and liquid–liquid interfaces. This is the first assembly process known to be shared by two peptidomimic families of molecules with crucial backbone differences. A series of AFM, SEM, TOF-SIMS, FTIR, X-ray crystallography, counterion screening experiments, QM calculations, and MD simulation studies identified that IFC is based on the assembly of single monomer layers with alternating molecular orientations, which results in bilayers of unit thickness 1.2–1.6 nm consisting of internal hydrophobic planes and ionic interfaces cocrystallized with halide salt ions. The assembly is underpinned by, paradoxically, the dynamic freedom of attached side chains, especially those of aliphatic designs. Growth of these bilayers then fills entire interfaces, limited only by the size of the container. The fundamental observation of the interface-filling nanostructures and the simplicity of the monomer chemistry involved suggest that IFC may have applications in the convenient formation of interface-sealing supramolecular barriers and, more broadly, tunable 2D layered materials.

## Introduction

Supramolecular assemblies, particularly those derived from peptides and peptidomimics, are of considerable interest for applications from therapeutics to advanced materials.^1−5^ Typically, the assembling molecules are amphiphilic and contain a balance of hydrophilic and hydrophobic chemical functionalities. In the context of peptides, the latter are often installed either as side chains of, e.g., phenylalanine (F), tyrosine (Y) and tryptophan (W),^6−11^ or as terminal functionalizations, e.g., naphthyl, benzyl and fluorenylmethoxycarbonyl (Fmoc) moieties.^12−14^ In both cases, the associated aromatic structures, being rigid and planar, lend an obvious directionality in their π–π stacking and dispersion interactions. A well-known example is the formation of highly rigid nanotubes from the FF dipeptide,^15^ which has inspired myriad self-assembly studies.^16,17^ Our mapping of the entire di- and tripeptide sequence spaces had further demonstrated that peptides containing aromatic dyads are particularly prone to self-assembly.^18,19^ Aromatic groups are also commonplace in other self-assembling molecules, and can enforce ordering even in achiral systems such as *N*-substituted glycine “peptoids”.^20−23^ Additionally, the strong organizing effects of aromatic groups can in many cases induce crystallinity, e.g., peptoid nanosheets.^24−28^

Strong assembly stabilization can however be difficult to reverse, consequently presenting challenges in designing dynamic systems and in managing material life-cycles. This motivated us to explore assemblies with deliberate exclusion of aromatic amino acids, yielding several novel surface-active and emulsion stabilizing tetrapeptides containing the aliphatic amino acids leucine (L), isoleucine (I) and valine (V).^29^ Nature, in fact, provides intriguing examples in using these aliphatic amino acids as well as alanine (A) for controlling self-assembly or folded structures that are stable but more dynamic and adaptive. The aliphatic side chains are rich in −CH_2_– rotors and the number of these methylene groups controls steric packing and strength of stabilizing dispersion forces.^30^ Thus, aliphatic side chains can enable residue packing effectively and are commonly found in critical secondary structure motifs.^31^ A prominent example is the α-helical DNA-binding “leucine zipper”.^32^ In antimicrobial peptides, leucine also promotes helical secondary structure, and substitution with alanine or a peptoid analogue may reduce helicity (and hemolytic activity).^33,34^ In other cases, aliphatic side chains are associated with self-assembly mechanisms similar to amyloid core sequences from degenerative disease proteins,^35^ and β-sheet secondary structures can be promoted in the order L/V > A > G^36^ for tuning the rigidity, stability and kinetic trapping of synthetic assemblies.^37,38^ A range of aliphatic di- and tripeptide designs have hence been demonstrated,^39−48^ including crystalline nanotubes (e.g., VX dipeptides, where X = A, V, I)^49^ crystals of heterochiral leucine-based dipeptides incorporating water channels and hydrophobic layers.^47^ The formation of bulk crystals from aliphatic amino acid monomers is furthermore long observed.^50−53^

As triggers to control assembly and crystallization, temperature and pH changes are common. However, modification of solvation in minimalist peptide and peptoid assembly has received less attention. We have however shown that an acetylated FF peptoid analogue may form stacked nanosheet plates or crystalline needles depending on whether water is used to induce precipitation from an acetonitrile stock solution (i.e., kinetic assembly) or DMSO is added to a water based solution to slow evaporation (i.e., thermodynamic assembly).^21^ We have also recently shown that solvent modification can be used to sensitively explore chemical libraries, e.g., Fmoc-A but no other aliphatic residue substitutions formed crystals in mixed water/THF, demonstrating how larger aliphatic side chains may compete with Fmoc directed assembly.^54^ Elsewhere, Erdogan et al. demonstrated assemblies ranging from plates to wires from VA and AV dipeptides by adjusting the addition of pyridine and 2-propanol in their system.^55^ These studies and others^46,56,57^ highlight the importance of taking a more holistic solvent plus solute view of assembly.

Air–liquid, liquid–liquid, and liquid–solid phase boundaries arise in many chemical contexts. These interfaces themselves may direct the formation of structures not otherwise accessible and permit additional control over material functionality. Classic examples include lipid bilayers,^58^ Langmuir–Blodgett films^59^ and self-assembled monolayers.^60^ Other examples include peptoid nanosheet assembly at air- and oil–water interfaces^61,62^ and the stabilization of emulsions by peptide self-assembly.^7,29,63^ However, overall, interfacial peptide assembly and crystallization have been less explored than solution phase systems.

**Scheme 1:**
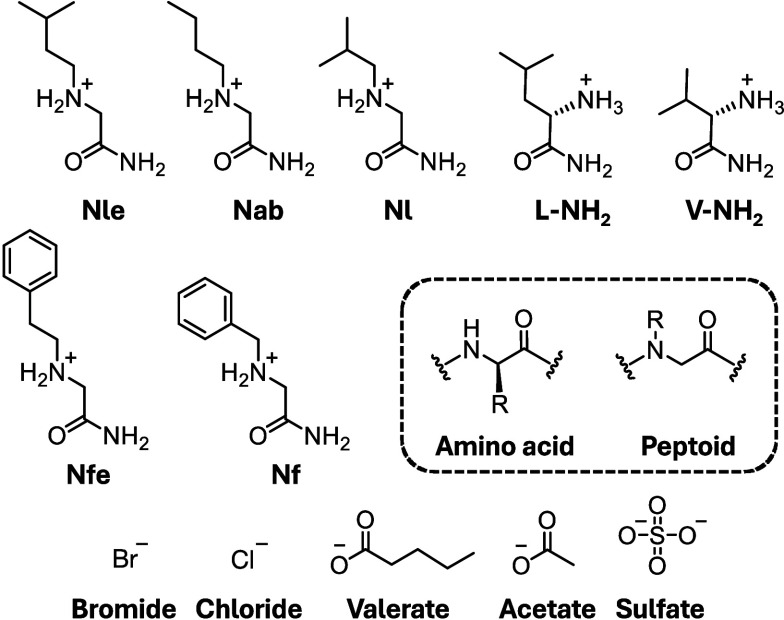
Peptoid and Amino Acid Amides and Counterions Studied in This Work The inset shows the structural distinction between an amino acid and its peptoid counterpart. In our nomenclature,^65^ peptoids are named firstly after the single letter code of the most closely related amino acid (e.g., Nl is a direct analog of L/leucine) and secondly after specific modifications (e.g., Nle is the ethyl side chain analog of L, Nab is the butyl analog of A/alanine, etc.).

In this article we focus on the role of aliphatic side chains in the formation and stabilization of interface-spanning crystalline sheets at liquid interfaces in a process we term interfacial crystallization (IFC). We first observed IFC during recrystallization of halide salts of a set^64^ of peptoid monomer amides with aliphatic and aromatic side chains. The interfacial sheets were observed to grow and fill entire cross sections of solution containers. We studied different peptoids and anions, and compared the results with key amino acid analogs (i.e., amino acid amides) (Scheme 1). The results provided generalizable molecular design insights for IFC and potential mechanistic explanations for this growth process. These were interrogated by a comprehensive study using scanning electron microscopy (SEM), atomic force microscopy (AFM), attenuated total reflection Fourier transform infrared (ATR-FTIR) spectroscopy, time-of-flight secondary ionization mass spectrometry (TOF-SIMS), reverse phase high performance liquid chromatography (RP-HPLC), quantum mechanical (QM) calculations, molecular dynamics (MD) simulations, single crystal X-ray crystallography, and solubility and salt exchange experiments.

## Results and Discussion

### IFC at Air–Liquid and Liquid–Liquid Interfaces

**Figure 1:**
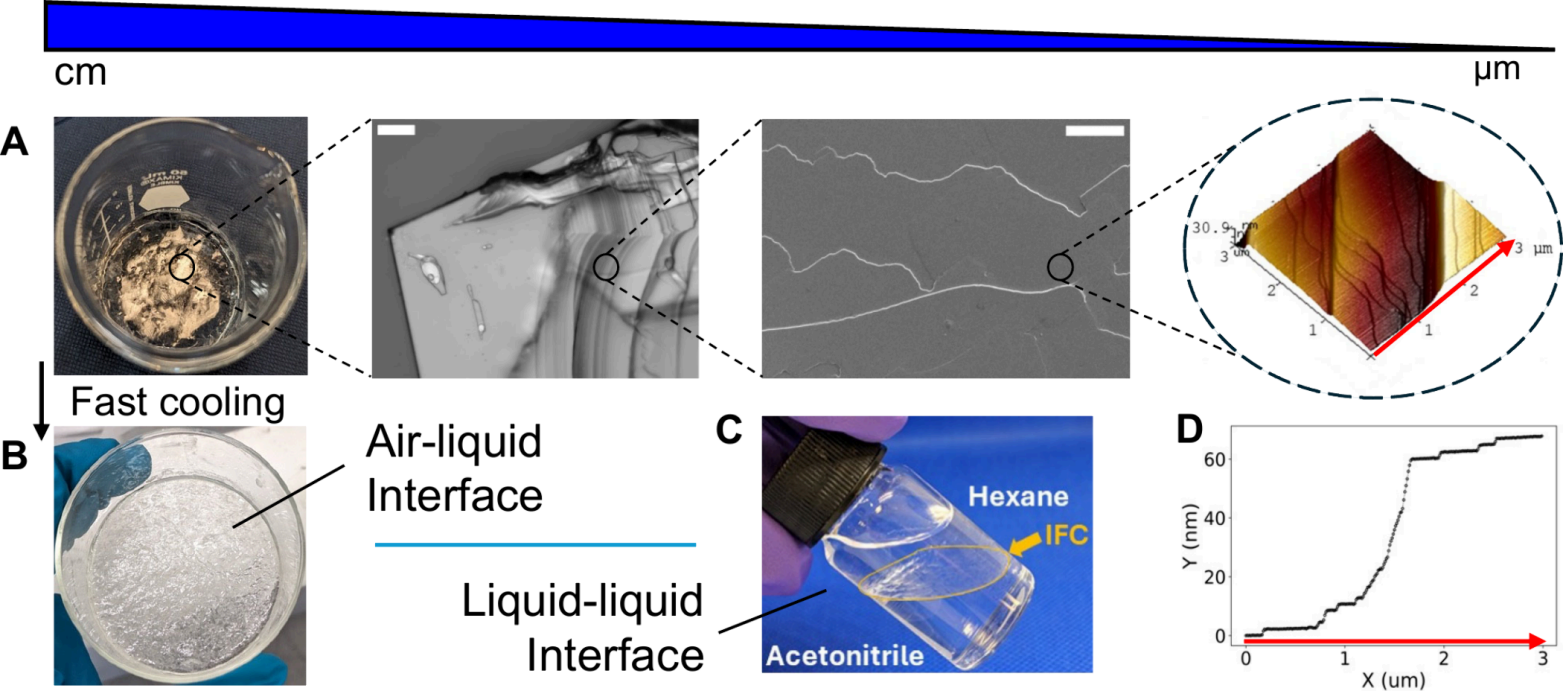
(A) 2D plane-filling interfacial crystal of Nle.HBr showing ordered structures at multiple length scales—from left to right: photograph of IFC spanning the surface of a 2:1 ACN:MeOH solution in a 50 mL beaker, with its crystalline thin-sheet nature evident from the almost uniform reflection of ambient light except around patches where slight buckling caused off-angled reflections and hence a darker appearance; optical micrograph (scale bar = 100 μm) showing extended crystalline edges and microscale terraces; SEM image (scale bar = 40 μm) showing curved microscopic terraces; and AFM image showing a hierarchy of nanoscale steps. (B) Interfacial assembly of Nle.HBr upon faster cooling led to a more amorphous but nonetheless space-filling surface. (C) IFC formed at the liquid–liquid interface of hexane/ACN:MeOH. (D) Line profile from the lower right edge of the AFM image shown directly above (red arrow), illustrating both a hierarchy of “unit” step heights 1.5–1.6 nm and larger terrace steps.

We initially discovered IFC for Nle, a peptoid monomer amide and leucine analog with a branched aliphatic side chain (Scheme 1; see caption for monomer naming convention). Figure 1 shows that when a solution of Nle bromide salt (i.e., Nle.HBr) in warm 2:1 v/v acetonitrile (ACN):methanol (MeOH) (T ≥ 50 °C) was left to slowly cool and evaporate, e.g., in a beaker covered with watch-glass, floating, transparent, crystalline sheets formed at the air–liquid interface. These ultimately fuse to form large structures that fill the liquid surface, e.g., across ∼15.5 cm^2^ for a 50 mL beaker (Figure 1A and Video S1). More rapid cooling and evaporation in an uncovered setup produced interfacial structures that appeared more disordered, though the interface was similarly filled (Figure 1B). The process was analogous to conventional recrystallization, except that no bulk crystals were formed, and it was fully reversible by the addition of methanol and/or reheating. Crystalline sheets at a liquid–liquid interface may also be formed (Figure 1C)—when an ACN:MeOH solution was cooled in a sealed vial and hexane (an immiscible solvent with lower density) was added down the side of the container, an interface-filling crystal layer appeared at the ACN:MeOH–hexane liquid boundary. These crystals were qualitatively much thinner and less cohesive compared to those formed at the air–liquid interface. Thus, IFC appears to be a general phenomenon across chemical phase boundaries.

Optical microscopy, SEM, and AFM inspections of the Nle.HBr sheets revealed a layered crystalline structure (Figures 1A, S1–S9), as displayed by the straight domain edges of individual crystallites on the millimeter scale and a hierarchy of surface terraces that extend to the nanoscale. AFM further identified a discrete minimum terrace height of 1.5–1.6 nm (Figures 1D and S10–S11). Notably the terraces have curved edges which we propose to be indicative of an IFC growth pathway, in which relatively slow lateral growth of individual terraces gave rise to curved edges while successive layers grew on top of each other as monomers were added from the solution phase. Thus, formed at the liquid interface, individual crystallite plates fused and gave rise to the domain boundaries observed on an IFC sheet.

**Figure 2:**
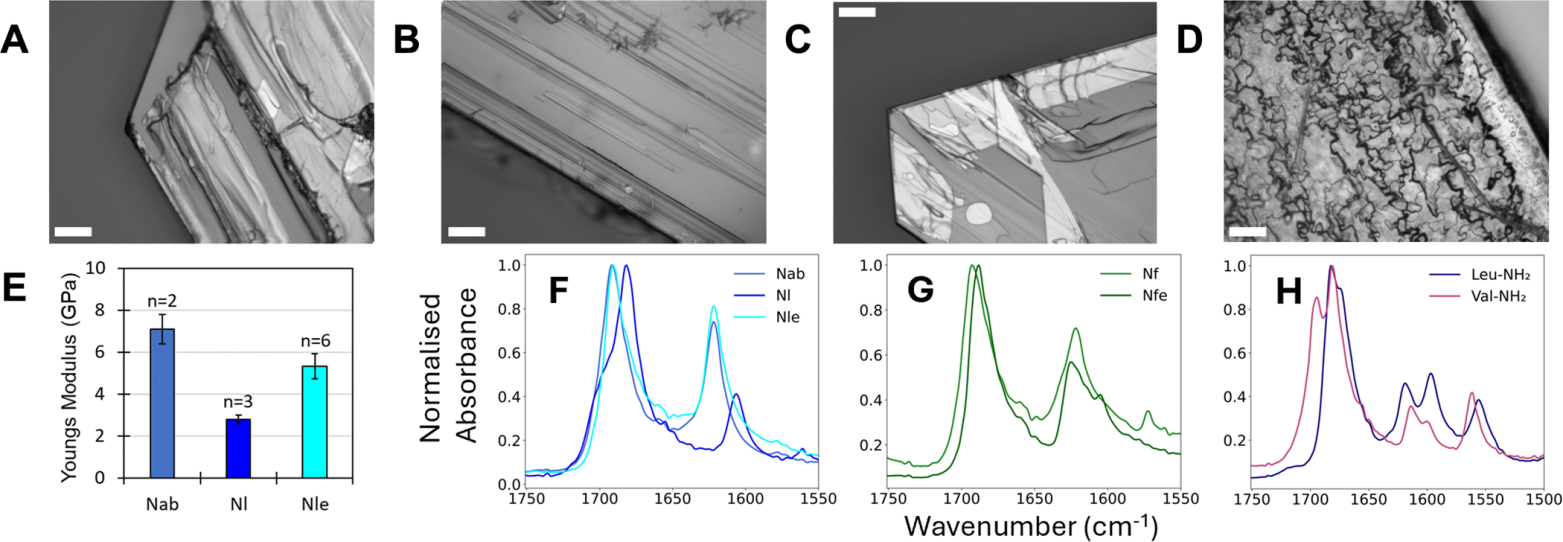
(A-D) Optical micrographs of crystals formed by bromide salts of Nab, Nf, Nfe and Nl respectively (scale bar = 100 μm). (E) AFM mechanical measurements for the aliphatic peptoid monomer amides. (F and G) ATR-FTIR spectra for, respectively, aliphatic and aromatic peptoid crystals showing the carbonyl stretch (∼1700 cm^–1^) and N–H bend (∼1620 cm^–1^) regions. (H) Corresponding ATR-FTIR spectra for IFC structures formed by chloride salts of L-NH_2_ and V-NH_2_.

We next prepared a set of related aliphatic and aromatic peptoids (Scheme 1) to better understand the IFC phenomenon. Together with Nle, Nab with a linear aliphatic butyl side chain as well as Nf and Nfe with aromatic side chains all exhibited IFC. Visually, all these species formed transparent crystal layers with well-defined edges (Figures 2A-D and S12–S20; see also Table S1 for ACN:MeOH mixing ratios). However, Nl, Nab’s branched isomer, only formed bulk crystals in solution with an aggregate-like appearance. These semicrystalline bulk crystals were visually opaque due to light scattering from a crenulated surface morphology on the microscale (Figure 2D and S21–S23), suggesting a different crystal structure (see further characterization below). In addition, IFC layers of the aromatic Nf and Nfe eventually settled into the solution, suggesting that the aromatic species gave rise to a denser crystal packing versus the aliphatic Nle and Nab. In fact, Nfe features prominently in peptoid assemblies,^21,26−28,66^ and one could speculate that a longer, more flexible ethylene side chain linkage may aid in optimizing structural packing (see QM/MD studies below).

### Nanoscale Surface Morphology, Mechanical Stiffness

Additional AFM imaging of Nab sheets showed, similar to Nle, a layered surface morphology composed of microscopic terraces (Figures 2A, S24) with minimum terrace heights of 1.2–1.4 nm (Figures S25–S26). In contrast, Nl bulk crystal surfaces showed no clear structure (Figure S27). AFM mechanical property measurements (Figure 2E) further showed that Nab and Nle exhibited similar Young’s moduli of 7 and 6 GPa, respectively. In contrast, the maximum modulus measured for Nl was lower at ∼3 GPa. In comparison, the moduli for the archetypical aromatic FF nanotubes and a partially aromatic tripeptide DYF-NH_2_ assembly we previously reported are higher at 19–30 GPa^67^ and ∼33 GPa,^8^ respectively. However, moduli lower than for the present aliphatic Nab and Nle structures have also been reported for several aromatic tripeptide fiber assemblies/crystals (e.g., ∼3 GPa for Boc-FF and for a series of amyloid fibrils).^8,68−70^ Taking together the mechanical strength and crystal surface morphology data, the IFC structure derived from Nab and Nle (and also Nf and Nfe) is clearly well-ordered and distinct from the bulk crystal of Nl.

### Hydrogen Bonding, Aliphatic Dispersive Contributions and Natural Amino Acid Amide Analogs

As an initial probe of the link between hydrogen bonding and structure, we performed ATR-FTIR spectroscopy. Focusing on the carbonyl stretch (∼1700 cm^–1^) and the amide N–H bending (∼1620 cm^–1^) regions, we found that all IFC structures belonging to Nle, Nab, Nf, and Nfe exhibited the hydrogen bonding bands at ∼1690 and ∼1620 cm^–1^ (Figures 2F,G and S28). These coincide with the amide I bands for antiparallel β sheet layers.^71^ However, we caution that the correlations between amide absorbances and secondary structure have not been established for peptoids. Regardless of structural assignment, for Nl, these bands were instead lower at ∼1680 and ∼1605 cm^–1^ (Figure 2F), indicating differences in hydrogen bonding.

We further compared the ratio of N–H bend to carbonyl stretch absorbances (Figure S29). This was ∼0.45 for Nl, but consistently higher at ∼0.6 for Nfe and at ∼0.8 for Nle, Nab, and Nf. We interpret this as indicating that the IFC structure leads to a distinct network of hydrogen bonding. Furthermore, the lower absorbance and wavenumber of the ∼1605 cm^–1^ peak in Nl versus the IFC molecules suggests that this network is less intense. This would moreover be consistent with the significantly higher stiffness measured for Nle and Nab, as discussed earlier (Figure 2E).

We next sought to assess the influence of dispersive contributions of aliphatic side chains on IFC propensity. First, it is well established that increasing the number of methylene units in an alkane generally increases the strength of dispersive attraction, and that branched alkanes have lower boiling points than their linear isomers.^72^ To corroborate this effect in peptoid monomer amides, we first performed RP-HPLC with a hydrophobic C18 column (Figure S30) and observed that Nl exhibited the lowest retention time and therefore the least dispersion interactions. Indeed, retention time increased from Nl to Nab, its linear isomer, and then to Nle, which has an additional methylene compared to Nl. Second, solubility tests in a series of ACN:MeOH mixing ratios showed increasing solubility in the order Nl < Nab < Nle as the fraction of the more hydrophobic ACN increased (Table S1). Calculated logP corroborate these observations, with hydrophobicity increasing also as Nl < Nab < Nle (Table S2). These measurements confirmed that Nl’s relatively low intramolecular dispersive interactions may decrease IFC propensity.

We also investigated leucine and valine amino acid amides (i.e., L-NH_2_, V-NH_2_) to further probe the role of dispersion interactions and to expand the generality of IFC. L-NH_2_ possesses the same side chain as the non-IFC forming Nl, and V-NH_2_’s branched side chain is even shorter (Scheme 1). Interestingly, both amino acid amides formed well-defined IFC sheets and terraces (Figures S31 and S32). Amino acids have a fundamentally different molecular arrangement from peptoids, with side chain attachment to a chiral backbone α-carbon. So, the observation of IFC for L-NH_2_ and V-NH_2_ suggests that sheet assembly is a general process for C-terminal amide peptidomimetic monomers. Of the two, L-NH_2_ formed IFC structures more readily, at rates similar to Nab and Nle. This confirmed that higher side chain dispersive attraction indeed promotes IFC but it is not on its own a limiting criterion. In fact, the calculated logP for V-NH_2_ is lower than for Nl (Table S2). At the same time, ATR-FTIR of L-NH_2_ and V-NH_2_ IFC crystals showed carbonyl stretch and N–H bend absorbances both similar and additional to the bands measured for the peptoid IFCs (Figure 2H). Thus, IFC may be supported by variations in the hydrogen bonding network. This contrasts with other systems, such as Fmoc-F and its peptoid analogs, for which the different morphologies observed were attributed to differences in hydrogen bonding.^73^

### QM/MD Studies of Side Chain Conformation

Since IFC may be supported by various levels of dispersion interactions and hydrogen bonding, we sought to investigate additional factors that may control it. Small modifications in side chain design are well-known to have profound impacts on molecular assembly.^74,75^ Moreover, the avoidance of steric side chain and backbone clashes has been considered to be a dominant factor influencing intrinsic β-sheet propensity of amino acid residues in proteins.^76^ We hypothesized that a similar mechanism is operative in IFC packing, whereby side chains and backbones are optimally segregated into hydrophobic and ionic layers. Specifically, we observed that the IFC peptoids Nab and Nle share a linear fragment of two consecutive −CH_2_– units that is lacking in Nl. This longer ethylene linkage may provide a degree of flexibility to optimize intermolecular interactions within a crystal lattice.

**Figure 3:**
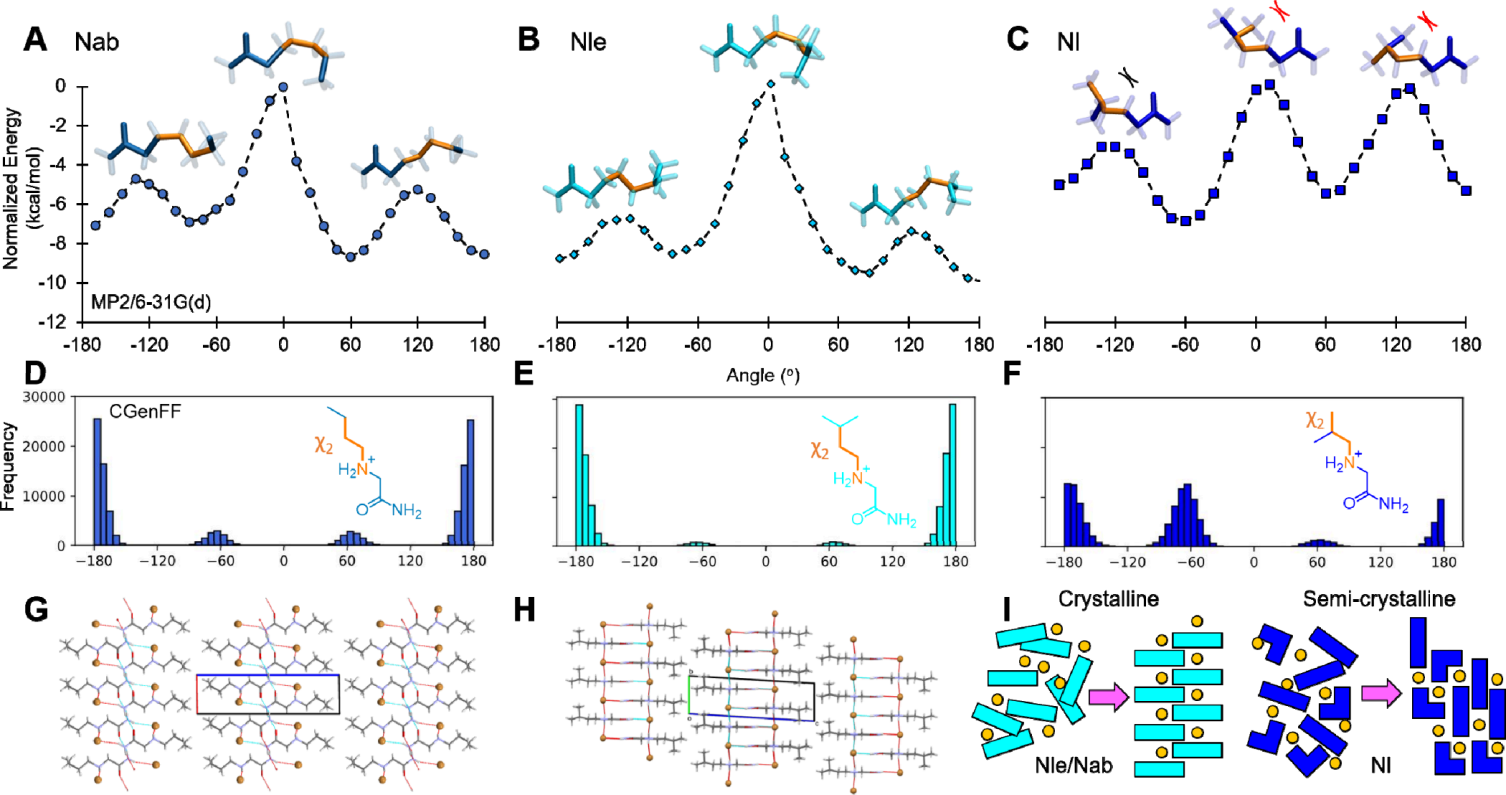
(A-C) Scans of χ_2_ torsion, defined as N–C_β_–C_γ_–C_δ_, at the MP2/6-31G(d) level of theory for Nab, Nle and Nl, respectively, with the conformer structures displayed at the energy maxima, highlighting the corresponding torsions in orange. In (C), red crosses indicate close contact between a methylene branch and the backbone, and the black cross indicates low energy contact with the C_β_ hydrogen. (D-F) MD samplings of χ_2_ torsion in Nab, Nle and Nl, respectively, corresponding to the energy landscapes shown in panels A to C. (G and H) Single crystal X-ray structures obtained for Nab.HBr and Nle.HBr, respectively. Crystallographic and refinement parameters are given in Tables S3 and S4. The c-axes shown in blue for both structures correspond to the long axis of the unit cell. (I) Schematics illustrating how conformational homogeneity (Nle and Nab IFC structures) and heterogeneity (Nl) give rise to different levels of ordering, and hence different assembly behavior and crystalline packings.

To assess this, we first performed χ_2_ torsion scans at the MP2/6-31G(d) level of theory for each of these aliphatic peptoid monomer amides (Figure 3A-C). Second, we corroborated the results with sampling of χ_2_ in 100 ns MD simulations (Figure 3D-E) (in ACN with chloride anions; bromide parameters were not available; see also next section on anion experiments). The χ_2_ torsion scans showed that all species displayed conformational energy minima at roughly ±60° and ±180°. However, the energy barriers were higher for Nl, especially away from the +60° minimum, while the energy profiles were more symmetric for both Nab and Nle. Moreover, the global minimum was −6.88 kcal/mol for Nl, while that of Nab, its structural isomer with a linear side chain, was lower at −8.66 kcal/mol.

The explanation lies in the proximity of Nl’s side chain’s terminal isopropyl branched unit to the backbone—significant energy penalties in the QM torsion scan were observed when the terminal −CH_3_ methylene branches and the backbone were close at 0° and 120° (clashes indicated by red crosses in Figure 3C). In comparison, this barrier was significantly reduced for the C_β_ proton at the branch point (black cross, Figure 3C). Consequently, different conformer populations would exist in Nl’s asymmetric χ_2_ energy landscape, as corroborated by the asymmetric sampling observed in MD (Figure 3F). This was broadly speaking not the case for Nab and Nle, for which the consecutive −CH_2_– units closest to the backbone interacted in a lower energy and roughly symmetric manner. This enabled sufficient spatial separation between the side chain and the backbone, lowering the −60° ↔ −180° and 60° ↔ 180° transition energies regardless of the terminal branching in the case of Nle (Figure 3A and B). MD simulations also captured this effect, as reflected in the broadly symmetric sampling for Nab and Nle, with a dominant ensemble at ±180° for both species (Figure 3D and E).

The torsion χ_2_, measured as N_ter_–C_α_–C_β_–C_γ_, was distinct for V-NH_2_ and L-NH_2_ side chains. Interestingly, V-NH_2_ shared similarities with Nl, suggesting that this profile is characteristic of a backbone-adjacent *ipso* group. Torsional sampling from MD simulations revealed heterogeneity for both species (Figures S33 and S34), despite IFC formation occurring in both cases. We attribute this to the pseudo-orthogonal positioning of the side chain relative to the backbone, which decouples conformational specificity from IFC formation. Based on these results we believe the formation of an ordered ionic salt layer becomes independent of side-chain conformation for this structural configuration.

The side chain conformational freedom requirement demonstrated above may also inform other examples of molecular assembly. For example, substitution of aromatic Nfe residues in peptoid nanosheet forming sequences with aliphatic residues showed that the structure with Nl was unstable.^77^ This was attributed to Nl’s low hydrophobicity but, in light of our findings, a contributing factor may have been Nl’s relatively inflexible side chain, which could have disrupted packing and hence assembly.

### X-ray Crystallography

To elucidate the internal structure of IFCs in detail and corroborate the QM/MD and other results, we obtained single crystal X-ray diffraction structures for Nle.HBr and Nab.HBr. The structures were found to be largely homologous and were composed of alternating hydrophobic and interdigitated ionic interfaces (Figure 3G and H, Tables S3 and S4). The hydrophobic interfaces were composed of opposing arrays of aliphatic side chains generally pointed away from the ionic backbone part of the monomers, reminiscent of bilayers. The ionic interfaces consisted of a 2:1 ratio of coordinations between bromide anions and N-terminal amines and between the anions and amidated C-termini from adjacent monomers, which enabled a continuous linkage of interdigitated monomers. The structure is further reinforced by hydrogen bonding between consecutive layered C-terminal amide groups (Figure S35), corroborating the hydrogen bonding network indicated by FTIR (Figure 2F). The overall stacked/layered structure is also in agreement with the terraced morphology observed in microscopy images. Given the AFM-measured minimum terrace heights of 1.2–1.6 nm (Figures 1E, S10 and S22), comparison with the crystal structure suggests that a single terrace may correspond to a bilayer (see also TOF-SIMS measurements further below).

Moreover, our crystallography results identified χ_2_ ∼ 180° for both Nab and Nle, which corroborates the energy minima at the MP2/6-31G(d) level of theory and the highest occupancies sampled in MD simulations. In comparison, Nl assembly and crystallization would be impeded by conformational heterogeneity (Figure 3I), consistent with the less intense hydrogen bonding network and lower stiffness measured (Figure 2E-G). Overall, the crystal structure corroborates our hypothesis that the critical molecular design principle for IFC is a sufficient steric freedom of aliphatic side chains around the χ_2_ torsion.

Lastly, we note that the inclusion of the C-terminal amide in bromide coordination confirms that this modification is important for the structure of IFC sheets. In contrast, crystals of aliphatic amino acids exhibit “hydrophilic” layers.^50−53^ On the other hand, the IFC’s alternating hydrophobic/ionic layered structure is reminiscent of the solid surface-templated peptoid crystals reported by Ma et al.^78^ While their solid–liquid system represents another example of 2D interfacial crystal growth, that assembly required comparatively long 12-mer alternating anionic-aromatic sequences to preorganize side chain carboxylate–calcium coordination and aromatic interactions. Thus, comparison with that solid–liquid crystal further emphasizes the remarkable feature of IFC in availing “only” aliphatic side chains and monomeric building blocks to direct ordered nanoassembly.

### Experimental and MD Simulation Studies on Anion Selectivity, Migration and IFC Growth

Given IFC’s layered structure, we further hypothesized that ion migration to the interface via poor solvent affinity could control IFC growth. We therefore screened the interaction of Nle with different types of anions, thus further probing the generality of the IFC phenomenon. Moreover, we performed TOF-SIMS measurements to characterize the surface chemistry of the crystalline sheets.

**Figure 4:**
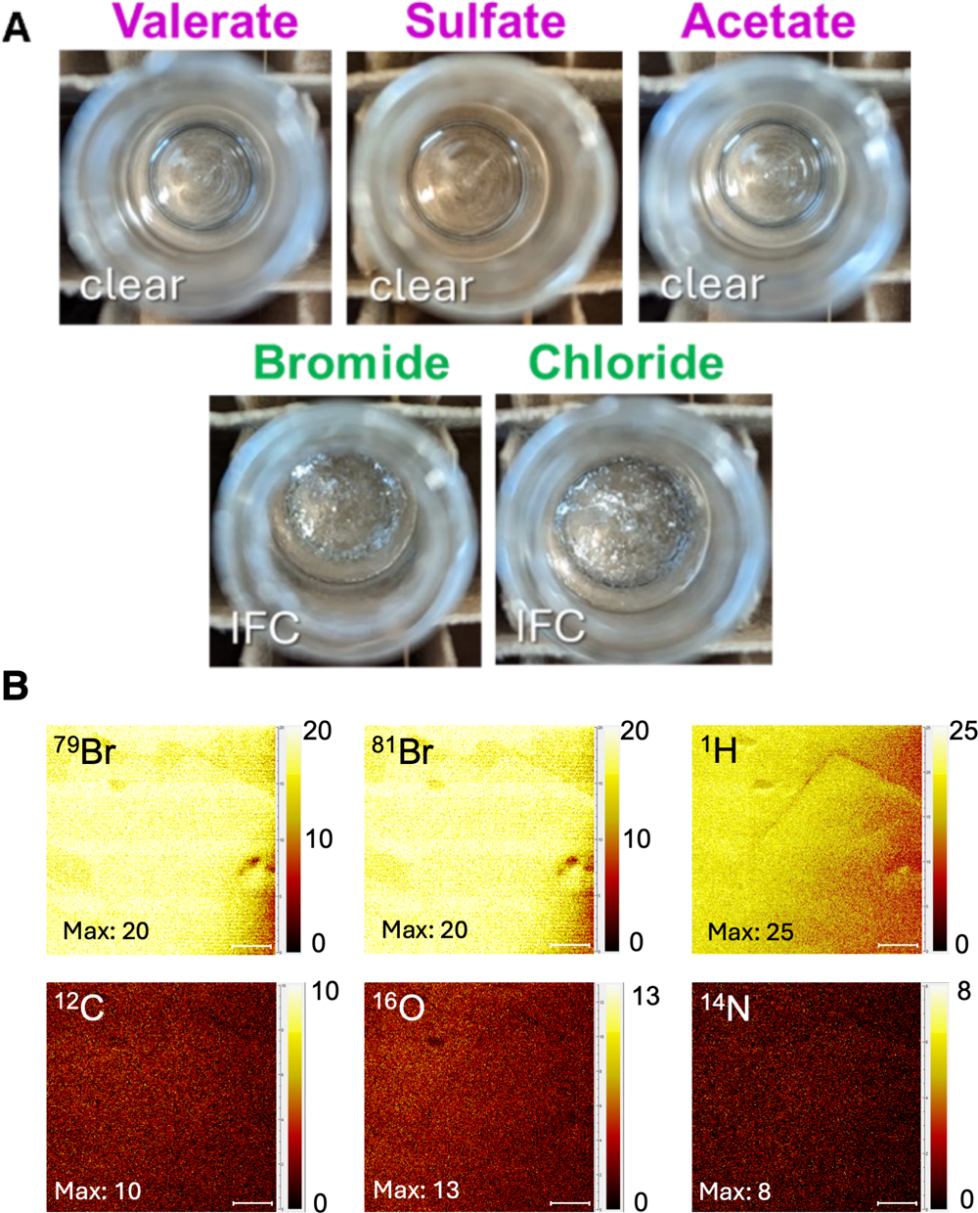
(A) Sulfate and the organic acids valerate and acetate did not give rise to IFC, whereas both bromine and chlorine halides ions formed IFC structures under the same conditions. (B) TOF-SIMS elemental maps of a representative area on a Nle.HBr IFC sheet sample showing substantially higher abundances of both bromine’s two stable isotopes ^79^Br and ^81^Br as well as atomic hydrogen (top row) were found within the first 1–2 nm of the crystal surface, as compared with atomic oxygen, carbon and nitrogen (bottom row) (scale bars = 100 nm).

We initially selected three anions with varying charge densities and aliphatic contents, namely the polyvalent inorganic sulfate and the organic anions valerate and acetate with, respectively, a longer butyl and a shorter methyl functionality (Scheme 1). No IFC was observed with any of these polyatomic anions (Figure 4A). Since the acetate and sulfate anions do not differ greatly in size from the bromide (radii of Br^–^ and SO_4_^2–^ are 198 and 242 pm, respectively, and acetate is in between),^79^ we attributed the lack of IFC with these polyatomic ions to their abilities to dissociate charge across multiple atoms and hence exhibit a greater affinity for ACN. The solubility of valerate was certainly higher than the acetate, and the differences in coordination geometry of the carboxylate and sulfate ions could also have been contributing factors. Thus, we further experimented with chloride anions and observed IFC crystalline interfaces (Figure 4A) as well as sheet surface morphologies (Figures S36–S42) equivalent to those formed from the bromide salt. Halide ions exhibit a high charge localization, which may be an important property giving rise to IFC.

To corroborate the notions that lower anion solvent affinity and higher charge localization may also control IFC propensity, we performed additional MD simulations to compare Nle’s interactions with acetate and chloride ions. Visual inspection of MD snapshots showed that in Nle.HCl simulations, a continuous and relatively compact structure would form (Figure S43A and B). In comparison, Nle and acetate ions formed smaller, disjointed aggregates (Figure S43C and D). Radial distribution functions (RDFs) (Figures S44 and S45) furthermore showed that, in the case of Nle.HCl, a ∼ 2:1 ratio in the numbers of chloride ions coordinating with the N-terminal amine and with the amidated C-terminus would emerge, which corresponds directly to the corresponding Nle X-ray crystal structure. In contrast, the ratio was ∼1:1 for the acetate, which is insufficient for formation of the interdigitated ionic interface of the IFC. These findings confirm that anion coordination and the balance of solvent affinity play crucial roles in IFC. Further MD studies based on L-NH_2_.HCl and V-NH_2_.HCl in an acetonitrile:vacuum system were also performed to simulate the air–liquid phase boundary and probe the IFC formation mechanism (Figure S46). Clusters were observed to assemble in the solution phase and migrate to the interface, suggesting that IFC could be initiated by this transfer process.

Lastly, we performed TOF-SIMS characterization on an Nle.HBr crystalline sheet as example (Figure 4B), under conditions that sputtered surface atoms only within a depth of 1–2 nm, matching the observed bilayer lamellar thickness. The results show that the crystal surface contained an abundant and homogeneous distribution of bromine atoms. Since the ionic interface is uniquely identified by bromine and that the current measurements highlight the elemental composition of only the topmost molecular layer, the data indicates IFC may preferentially terminate with this crystalline plane. While TOF-SIMS is known to give only qualitative results of elemental composition, considering the similar energies required to ionize bromine and carbon atoms for them to be detected (11.8 eV for bromine vs 11.3 eV for carbon),^80^ the higher intensities measured for ^79^Br and ^81^Br (present only at the ionic interface) compared to ^12^C (present at both the ionic and hydrophobic interfaces) supports that the ionic interface is exposed on the IFC sheet surface. This would be consistent with our hypothesis of a slow(er) growing ionic interface controlling IFC.

## Conclusion

We present the discovery of interfacial crystallization (IFC) that produces crystalline sheets spanning air–liquid and liquid–liquid interfaces. The interfacial sheets arise from a novel coassembly of halide ions and C-terminal amide monomers of peptoids and amino acids. IFC is distinct in both morphology and internal structure from commonly observed bulk amino acid crystals. Crystallization was found to be controlled by hydrophobic side chain interactions and by interfacial ion migration and coordination, resulting in alternating hydrophobic and ionic internal layers. This was confirmed by X-ray crystal structure determination and complementary experimental and computational studies with different anions. TOF-SIMS further identified an external ionic crystal surface, and AFM results were consistent with IFC formation through the growth of molecular “bilayers” of thickness 1.2–1.6 nm.

Our results further indicated that the molecular requirements of IFC should be generalizable to all aliphatic peptoid and amino acid amides with sufficiently large side chains. While IFC was also observed for aromatic peptoid monomers, their sheets eventually precipitated into the solution phase. This implied a denser packing, consistent with the propensity of aromatic residues to promote assembly, but also represented a drawback in preventing a sustained interfacial layer. ATR-FTIR and solubility measurements further identified that varying levels of peptoid or amino acid hydrogen bonding and dispersion interactions may support IFC. Importantly, QM/MD revealed that conformational flexibility in side chain χ_2_ torsion conferred by ethylene linkages in aliphatic peptoid side chains best promoted IFC. The understanding this observation could offer for past nanoassembly studies have been discussed.

Overall, IFC was shown to be a remarkable phenomenon that can reversibly fill air–liquid and liquid–liquid interfaces on the macroscale and that is constituted simply from peptoid *and* amino acid amide *monomers*. The fact that both kinds of monomers can share a common structuring process despite differences in backbone and side chain arrangements illustrates the fundamental novelty of IFC. The monomers also represent a diverse set of minimalistic peptidomimetic building blocks, making IFC a readily accessible mode of supramolecular structure fabrication. Our measurements moreover indicate high stiffness for the IFC sheets. Future work could focus on developing various applications, including its use as catalyst support (e.g., through cocrystallization) and the reversible partitioning of interfaces in nonequilibrium assembly systems.

## Data Availability

All data described in this publication are openly available from the University of Strathclyde KnowledgeBase at http://doi.org/10.15129/a42cd09c-9615-4708-895c-b53879ef0b52.
