# Supplementary material for: 2D Interfacial Crystallization Stabilized by Short-Chain Aliphatic Interfaces
Source: Langmuir. 2025 Mar 11;41(11):7376–85. doi: 10.1021/acs.langmuir.4c04718 (PMC11948478; doi:10.1021/acs.langmuir.4c04718)
Supplement: Supplementary file 1 — la4c04718_si_001.pdf [file la4c04718_si_001.pdf]

Supplementary Information

2D Interfacial Crystallization Stabilized by Short-Chain Aliphatic Interfaces

Hamish W. A. Swanson,[1,2] Kenny Barriales,[2,3,4] Emmet A. Sherman,[2] Tai-De Li,[2,5] Alan R. Kennedy,[1] Tell Tuttle,[1*] Rein V. Ulijn,[3,4,6*] and King Hang Aaron Lau[1*]

[1] Department of Pure and Applied Chemistry, University of Strathclyde, 295 Cathedral Street, Glasgow G1 1XL, UK.

[2] Nanoscience Initiative at Advanced Science Research Center, The Graduate Center, The City University of New York, 85 Saint Nicholas Terrace, New York, NY 10031, USA.

[3] Department of Chemistry, Hunter College, The City University of New York, 695 Park Avenue, New York, NY 10065, USA.

[4] Ph.D. Program in Chemistry, The Graduate Center, The City University of New York, 365 5th Ave, New York, NY 10016, USA.

[5] Department of Physics, The City College of New York, The City University of New York, 160 Convent Avenue, New York, NY 10031, USA.

[6] Advanced Science Research Center, The Graduate Center, The City University of New York, 85 Saint Nicholas Terrace, New York, NY 10031, USA.

CORRESPONDING AUTHORS

King Hang Aaron Lau – Department of Pure and Applied Chemistry, University of Strathclyde, 295 Cathedral Street, Glasgow G1 1XL, UK; orcid.org/0000-0003-3676-9228;
Email: aaron.lau@strath.ac.uk
Tell Tuttle – Department of Pure and Applied Chemistry, University of Strathclyde, 295 Cathedral Street, Glasgow G1 1XL, UK; orcid.org/0000-0003-2300-8921;
Email: tell.tutle@strath.ac.uk
Rein V. Ulijn – Advanced Science Research Center, The Graduate Center, The City University of New York, 85 Saint Nicholas Terrace, New York, NY 10031, USA; Department of Chemistry, Hunter College, 695 Park Avenue, New York, NY 10065, USA; Ph.D. Program in Chemistry, The Graduate Center, The City University of New York, 365 5th Ave, New York, NY 10016, USA; orcid.org/0000- 0002-7138-1213;
Email: rulijn@gc.cuny.edu

Contents

Table of Videos **3**

Table of Figures **3**

Table of Tables **5**

I. SUPPLEMENTARY DATA (Video S1, Figures S1 to S46, Tables S1 to S4) **6**

I.1. Video S1 of Nle IFC formed on the surface of 2:1 v/v acetonitrile (ACN):methanol (MeOH) 6

I.2. Nle IFC Crystal Morphologies 7
Bright Field Optical Microscopy Characterization for Nle.HBr Samples 7
Scanning Electron Microscopy (SEM) Characterization for Nle.HBr samples 8
Atomic Force Microscopy (AFM) Characterization for Nle.HBr samples 10

I.3. Crystal Morphology Characterization by Bright Field Optical Microscopy of Nab, Nf, Nfe IFC Crystals and Nl non-IFC Crystals 13
Representative optical microscopy images of crystal surfaces obtained for 3 independent replicates of Nab.HBr IFC formation experiments. 13
Representative optical microscopy images of crystal surfaces obtained for 3 independent replicates of Nf.HBr IFC formation experiments. 14

Representative optical microscopy images of crystal surfaces obtained for 3 independent replicates of Nfe.HBr IFC formation experiments. 15
Representative optical microscopy images of crystal surfaces obtained for 3 independent replicates of Nl.HBr crystallization experiments. 16

I.4. AFM Topography Measurements of Nab IFC Crystal and Nl Bulk Crystal 17
AFM Topography Imaging of Nab.Hbr Samples 17
AFM Step Height Characterization for the Nab.Hbr IFC Sample Surface 17
AFM Topography Imaging of Nl.Hbr Sample 19

I.5. ATR-FTIR Measurements 20

I.6. Hydrophobicity Comparisons of Aliphatic Peptoid Amide Monomers 22
Solvent Compositions for IFC Formation from Peptoid Amide Monomers 22
RP-HPLC and Solubility Experiments 23
LogP Calculation 24

I.7. Amino Acid Amide IFC Crystal Morphologies Characterized by Optical Microscopy 25

I.8. $N_{ter} – C_{\alpha} – C_{\beta} – C_{\gamma}$ Torsion Scans 26

I.9. Single Crystal X-Ray Crystallography Results 27

I.10. Comparison of the Morphologies of Nle Crystals Formed with Chloride and Bromide Salts, as Characterized by Optical Microscopy 30

I.11. Molecular Dynamics Solution Measurements 31

I.12. Molecular Dynamics Simulations at an Acetonitrile:Vacuum Interface 34

II. EXPERIMENTAL (including Tables S5 and S6, and Figures S47 to S53) 36

II.1 Materials and Synthesis of Peptoid Amide Monomers 36

II.2 Crystal Preparation 36

II.3 Experiments to Screen Anion Type 36

II.4 Characterization 38
Nuclear Magnetic Resonance (NMR) Spectra for Purified Peptoid Amide Monomers 38
Optical Microscopy 41
Scanning Electron Microscopy (SEM) 41
Atomic Force Microscopy (AFM) 41
Time of Flight Secondary Ionization Mass Spectrometry (TOF-SIMS) 41
Molecular Dynamics Simulations 41
Quantum Mechanical Calculations 42
Attenuated Total Reflectance Fourier Transform Infrared Spectroscopy (ATR-FTIR) 42
HPLC Measurements 42
Single Crystal X-Ray Diffraction. 42
AFM Step Height Estimation 42

III. REFERENCES 45

Table of Videos

Video S1 of Nle IFC formed on the surface of 2:1 v/v acetonitrile (ACN):methanol (MeOH) Figure S1. Nle.HBr sheet surface – experiment 1. 7

Table of Figures

Figure S1. Nle.HBr sheet surface – experiment 1....... 7
Figure S2. Nle.HBr sheet surface – experiment 2....... 7
Figure S3. Nle.HBr sheet surface – experiment 3....... 7
Figure S4. Representative SEM image of a Nle.HBr IFC sheet surface showing curved steps corresponding to the largest scale terrace features found in AFM measurements. 8
Figure S5. A higher magnification SEM image of the Nle.HBr crystal shown in Figure S1 above. 8
Figure S6. SEM image of another area of the Nle.HBr crystal surface showing analogous surface terrace structures as Figures S1 and S2....... 9
Figure S7. SEM image of selected areas of the Nle.HBr crystal surface showing straight steps. Thus, growing terrace fronts were not exclusively curved. However, higher magnification AFM images (Figure S6) shows that finer scale curved terrace edges on the submicron scale may co-exist with these microscopic straight edges. 9
Figure S8. Representative 20 x 20 µm AFM measurement of a Nle.HBr IFC sheet surface showing straight terraces with step heights on the tens of nanometer scale. However, close inspection of the terrace planes show finer scale curved terrace edges (see below). 10
Figure S9. A higher magnification 3 x 3 µm AFM measurement of the Nle.HBr crystal surface showing discreet curved terraces. Note the larger scale straight terrace edge co-existing at the right corner of image. 10
Figure S10. A) Histogram of IFC terrace step heights measured for the Nle.HBr sample, based on the AFM topography image shown in Figure S9. The data is compiled from step heights identified from line profiles shown in Figure S11. B) Recalculated step heights obtained after correcting for an artefact induced by routine AFM topography data processing. See further explanation associated with Figure S11 and the last section of the Experimental on "AFM Step Height Estimation"....... 11
Figure S11. 10 line profiles measured on an AFM image taken for Nle.HBr (Figure S9)....... 12
Figure S12. Nab.HBr sheet surface – experiment 1....... 13
Figure S13. Nab.HBr sheet surface – experiment 2....... 13
Figure S14. Nab.HBr sheet surface – experiment 3....... 13
Figure S15. Nf.HBr sheet surface – experiment 1. 14
Figure S16. Nf.HBr sheet surface – experiment 2. 14
Figure S17. Nf.HBr sheet surface – experiment 3. 14
Figure S18. Nfe.HBr sheet surface – experiment 1....... 15
Figure S19. Nfe.HBr sheet surface – experiment 2....... 15
Figure S20. Nfe.HBr sheet surface – experiment 3....... 15
Figure S21. Nl.HBr crystal....... 16
Figure S22. Nl.HBr crystal....... 16
Figure S23. Nl.HBr crystal....... 16
Figure S24. Representative 3 x 3 µm AFM image of Nab.Hbr IFC crystal surface showing terraced layers with well defined edges. 17
Figure S25. A) Histogram of IFC terrace step heights measured for the Nab.HBr sample, based on the AFM topography image shown in Figure S24. The data is compiled from step heights identified from line profiles shown in Figure S26. B) Recalculated step heights obtained after correcting for an artefact induced by routine AFM topography data processing. See further explanation associated with Figure S11 and the "AFM Step Height Estimation" section in II.4 Characterization. The mean and standard deviations are conserved before and after the correction....... 17
Figure S26. 10 line profiles measured on an AFM image taken for Nab.HBr (Figure S24). scan measurements taken for Nab.HBr, illustrating clear steps....... 18
Figure S27. 3 x 3 µm AFM measurement of a Nl.HBr bulk crystal surface showing a lack of well-defined structure, consistent with optical microscopy observations (See S19). 19
Figure S28. a) Normalized ATR-FTIR measurements for a second batch of crystals formed from all five peptoid amide monomers included in the study (first set of results shown in Figure 2 of the main text). b) Subset of the data showing only the results for aliphatic residues—both Nle and Nab formed IFC structures and Nl did not. c) Normalized ATR-FTIR measurements for a *third* batch of crystals formed from the five peptoid amide monomers. d) Corresponding subset of the data showing only the results for aliphatic residues Nab, Nl, and Nle. All batches of Nl.HBr show a different pattern of amide I and II shifts (shoulder instead of peak at ~1690 cm^{-1} and peak at ~1605 cm^{-1} instead of ~1620 cm^{-1}) compared to the rest of the peptoids that underwent IFC....... 20
Figure S29. Amide II/I absorbance ratios averaged across three set of experiments for all peptoid measurements (original data shown in Figure 2 in the main text and in Figure S28 above). Clearly, the amide II/I ratio is substantially reduced for the Nl bulk crystals (~0.4) compared with all other species (0.65~0.82). The error bars show ±1 SD. 21
Figure S30. A) Plot of RP-HPLC retention time vs. the volume of methanol added to acetonitrile (ACN) in order to precipitate the dissolved aliphatic peptoid amide monomers (see data in Table S1). B, C, D) Original RP-HPLC chromatograms of Nl, Nab, and Nle monomers, respectively, with absorbances measured at 214 nm. 23

Figure S31. IFC formed by L-NH_2.HCl showing clearly defined terraced layers as found in the peptoid amide IFCs suggesting a shared growth mechanism. 25
Figure S32. IFC formed by V-NH_2.HCl also showing terraced layers though the crystals are less well defined than their leucine-amide counterparts. 25
Figure S33. N_{ter}–C_α–C_β–C_γ torsional scan for V-NH_2 at MP2/6-31G(d) level of theory and sampling frequency extracted from MD simulations. Interestingly, this shares similarities with Nl, suggesting that this profile is characteristic of a backbone-adjacent *ipso* group. MD sampling shows good agreement with QM energy minima obtained. 26
Figure S34. N_{ter}–C_α–C_β–C_γ torsional scan for L-NH_2 at MP2/6-31G(d) level of theory and sampling frequency extracted from MD simulations which are in good agreement. Notably the functional form of the torsion is distinct from that of the studied peptoid monomers and V-NH_2 while the IFC phenomena is conserved. This result suggests that for this amino acetimide salt sidechain confromation and IFC packing are decoupled. 26
Figure S35. Single X-ray diffraction structures of Nle.HBr (A) and Nab.HBr (B). The presence of intermolecular hydrogen bonding via the N-termini (blue) and 2:1 ionic coordination between anions and protonated amine/amide termini (red) show that both modes of intermolecular noncovalent interactions are important in the formation of IFC crystals. Furthermore, the extension of the sidechain with $\chi 2 \sim \pm 180^o$ supports the observed sampling in MD simulations for these species, as well as the energy minima in QM torsion scans (see Figure 3 in the main text). 29
Figure S36. Nle.HCl with polarised light 30
Figure S37. Nle.HBr with polarised light 30
Figure S38. Nle.HBr with polarised light 30
Figure S39. Nle.HCl with polarised light 30
Figure S40. Nle.HBr with polarised light 30
Figure S41. Nle.HCl with polarised light 30
Figure S42. Nle.HBr with polarised light 30
Figure S43. Final structures from two separate 100 ns MD simulations of Nle.HCl (A and B) and Nle.H-acetate (C and D) in ACN (snapshots obtained after centering and clustering). Nle molecules are those in blue, while green and pink beads correspond to chloride and acetate anions respectively. 31
Figure S44. Duplicate RDFs for Nle.H-acetate (A and B), Nle.HCl (C and D), and Nab.HCl (E and F). (G) Illustration of coordination centers about which RDFs are calculated for the Nle.HBr structure. 32
Figure S45. Radial distribution functions (RDFs) of chloride anion and N-centers in Nle peptoid monomer using our recently parameterized peptoid N-terminus partial charge model. A split coordination shell was observed for the N-terminal $NH_2(+)$ group which arises due to the electrophilic character of the adjacent $C_\alpha H_2$ unit, as obtained by fitting partial charges towards the reproduction of water binding energies in multiple interaction complexes. This result is of interest as it highlights the polarized nature of the $C_\alpha H_2$ unit in peptoid backbones, which is also observed *via* NMR measurements in D_2O solvent. 33
Figure S46. Example y/z perspectives of the acetonitrile:vacuum interface simulated for 250 ns in the NVT ensemble for L-NH_2.HCl, where constituent entities are represented by their collective center of mass (COM). From top to bottom, snapshots at increasing 50 ns intervals are shown. 34
Figure S47. ^1H NMR of.Nle.HBr (400 MHz, D_2O) δ (ppm): 0.87 (d, 6H), 1.54 (q, 2H), 1.62 (nonet, 1H), 3.06 (t, 2H) and 3.85 (s, 2H). 38
Figure S48. ^1H NMR of Nle neutral amine for salt screening experiments (400 MHz, d6-acetone) δ (ppm): 0.905 (d, 6H), 1.38 (q, 2H), 1.68 (nonet, 1H), 2.60 (t, 2H), 2.80 (s, 2H), 3.12 (s, 2H), 6.27 (s, 0.58) and 7.06 (s, 2H). (Note: peaks at g and h are interpreted as the N-terminal proton which is split between two conformations). 38
Figure S49. ^1H NMR of Nl.HBr (400 MHz, D_2O) δ (ppm): 0.95 (d, 6H), 1.99 (nonet, 1H), 2.89 (d, 2H) and 3.85 (s, 2H). (Note: inverted signal at 4.70 ppm is H_2O). 39
Figure S50. ^1H NMR of Nab.HBr (400 MHz, D_2O) δ (ppm): 0.87 (t, 6H), 1.34 (sextet, 2H), 1.63 (quintet, 2H), 3.02 (t, 2H) and 3.84 (s, 2H). (Note: 4.70 ppm shift is H_2O). 39
Figure S51. ^1H NMR of Nf.HBr (400 MHz, D_2O) δ (ppm): 3.84 (s, 2H), 4.24 (s, 2H) and 7.45 (m, 5H). (Note: peak at 4.70 ppm is H_2O). 40
Figure S52. ^1H NMR of Nfe.HBr (400 MHz, D_2O) δ (ppm): 3.01 (t, 2H), 3.33 (t, 2H), 3.85 (s, 2H) and 7.34 (dt, 5H). Note: inverted peak at 4.70 ppm is H_2O. 40
Figure S53. Illustration of the process for step-height estimation via AFM measurement, showing the Nle.HBr data as example. A) Line flatterning was applied to the original AFM data. B) Line profiles were then drawn using the native NanoScope software across the parts of the surface exhibiting terrance steps. Typically 10 profiles were obtained. C) An example of these traces from these step heights were calculated following steps 3 and 4 described in the text. 44

Table of Tables

Table S1. Specific solvent compositions for comparative crystallization study. See Experimental section on crystal preparation (II.3) for details on methodology. 22
Table S2. Calculated LogP Values for candidate IFC molecules 24
Table S3. Selected Crystallographic and Refinement Parameters for Nle.HBr. 27
Table S4. Selected Crystallographic and Refinement Parameters for Nab.HBr. 28
Table S5. Suppliers of amine starting materials 36
Table S6. Yields for Nle neutral amine work up. 36

I. SUPPLEMENTARY DATA (Video S1, Figures S1 to S46, Tables S1 to S4)

I.1. Video S1 of Nle IFC formed on the surface of 2:1 v/v acetonitrile (ACN):methanol (MeOH)

https://drive.google.com/file/d/1tSE1lAes4R8Fnzu80Y-OrNcbWXfqq7B7/view?usp=drivesdk

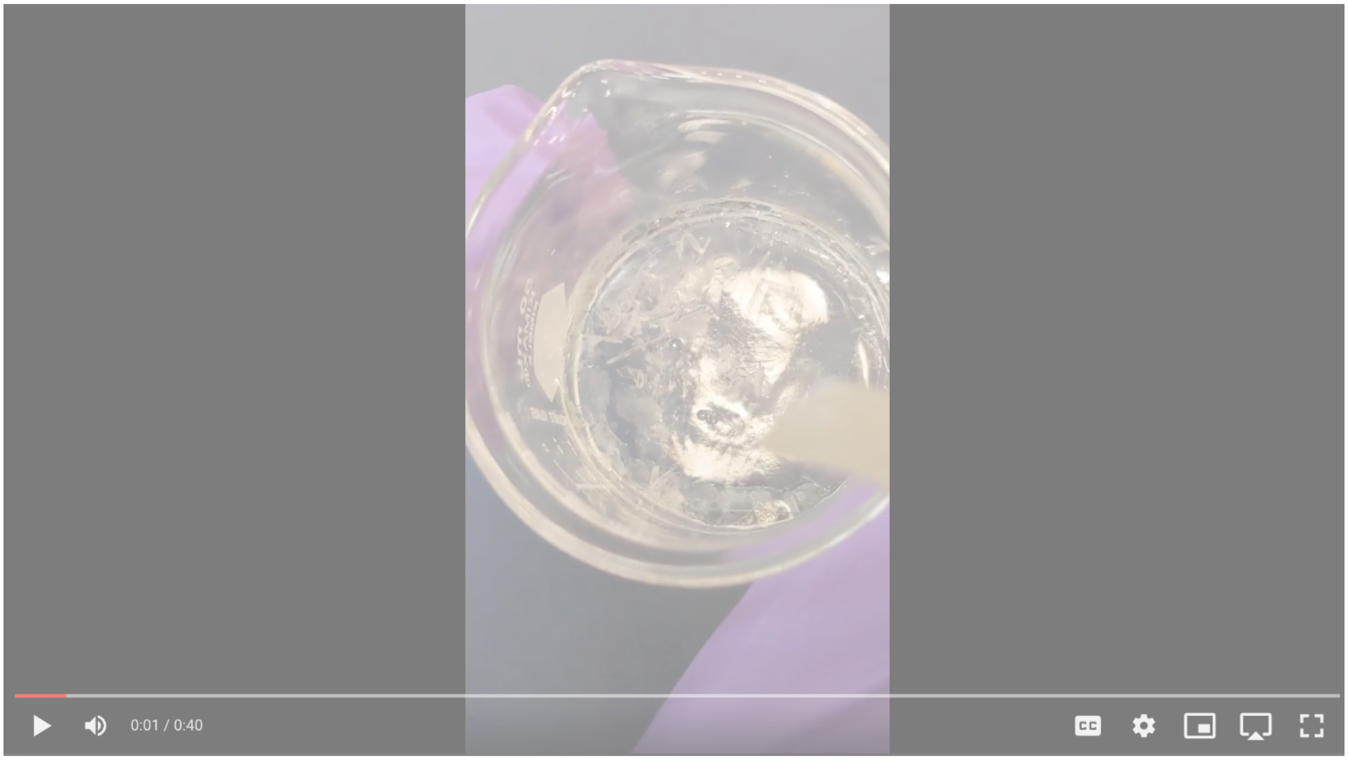

I.2. Nle IFC Crystal Morphologies

Bright Field Optical Microscopy Characterization for Nle.HBr Samples

Representative optical microscopy images of crystal surfaces obtained for 3 independent replicates of Nle.HBr IFC formation experiments.

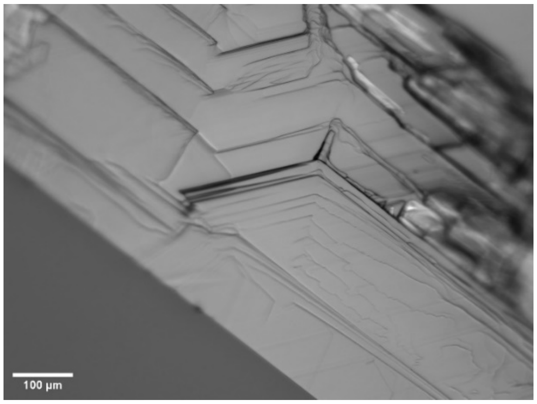

Figure S1. Nle.HBr sheet surface – experiment 1.

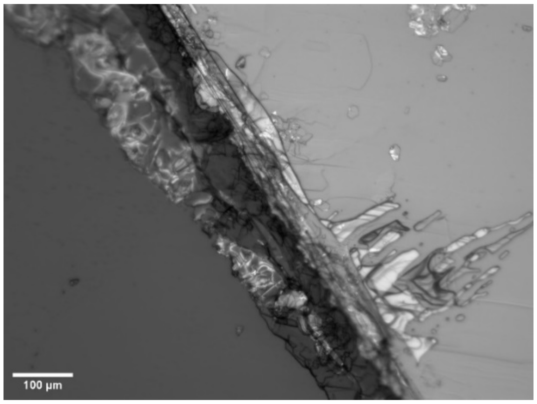

Figure S2. Nle.HBr sheet surface – experiment 2.

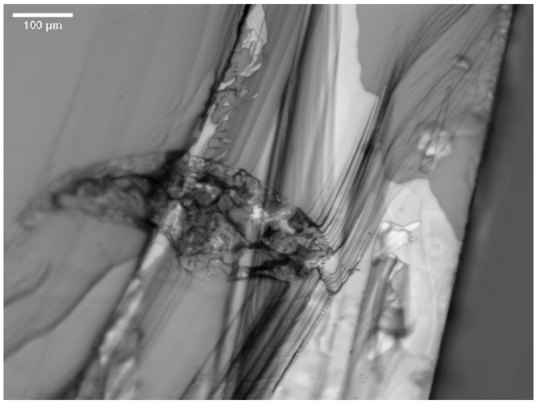

Figure S3. Nle.HBr sheet surface – experiment 3.

Scanning Electron Microscopy (SEM) Characterization for Nle.HBr samples

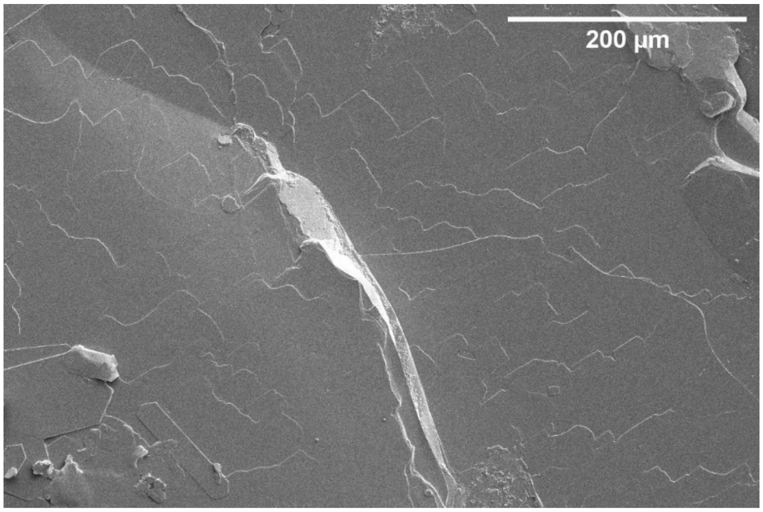

Figure S4. Representative SEM image of a Nle.HBr IFC sheet surface showing curved steps corresponding to the largest scale terrace features found in AFM measurements.

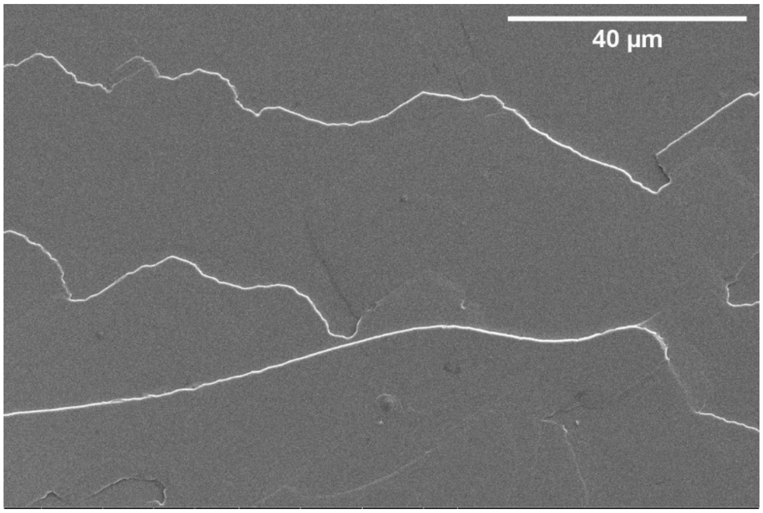

Figure S5. A higher magnification SEM image of the Nle.HBr crystal shown in Figure S1 above.

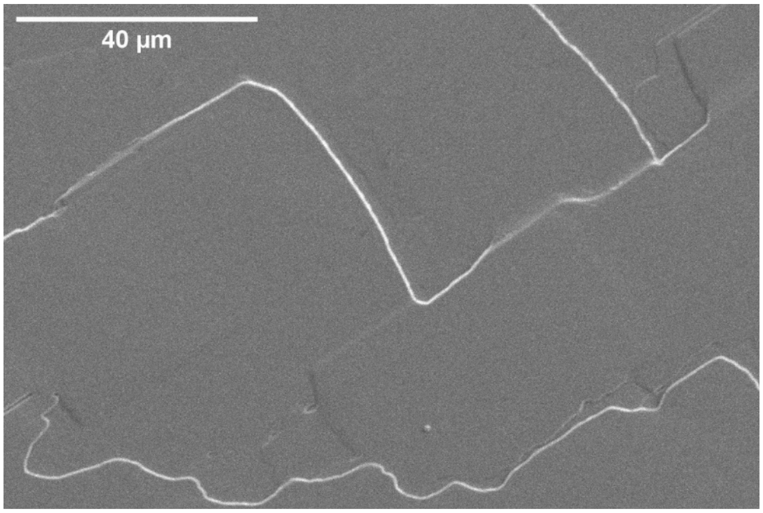

Figure S6. SEM image of another area of the Nle.HBr crystal surface showing analogous surface terrace structures as Figures S1 and S2.

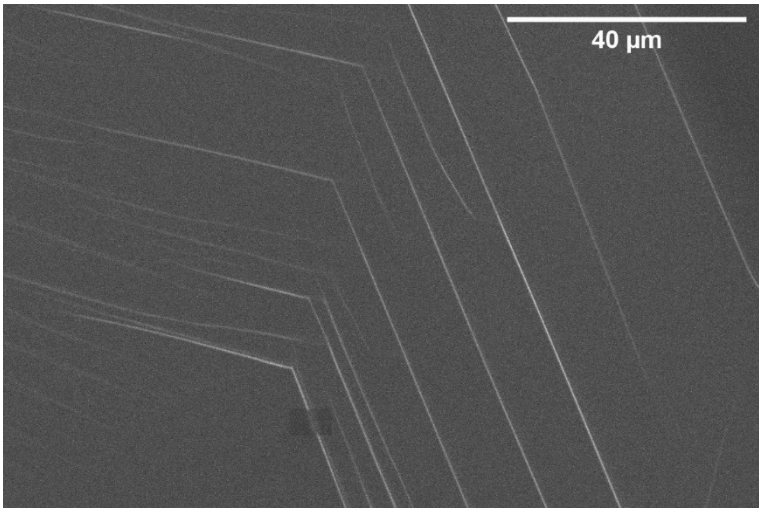

Figure S7. SEM image of selected areas of the Nle.HBr crystal surface showing straight steps. Thus, growing terrace fronts were not exclusively curved. However, higher magnification AFM images (Figure S6) shows that finer scale curved terrace edges on the submicron scale may co-exist with these microscopic straight edges.

Atomic Force Microscopy (AFM) Characterization for Nle.HBr samples

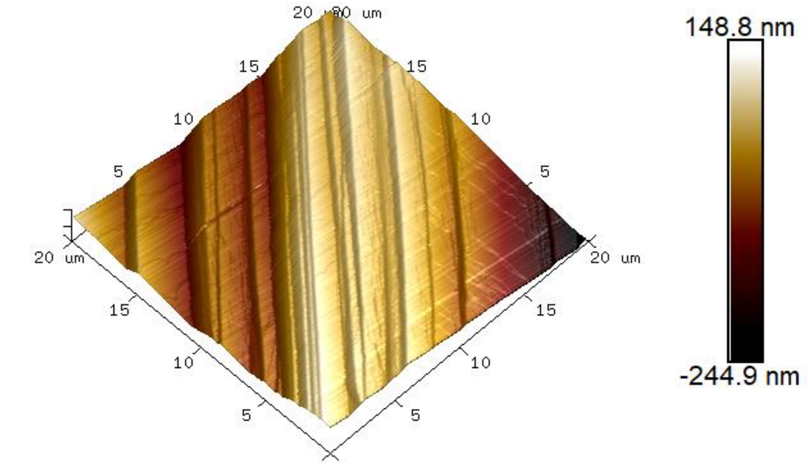

Figure S8. Representative 20 x 20 μm AFM measurement of a Nle.HBr IFC sheet surface showing straight terraces with step heights on the tens of nanometer scale. However, close inspection of the terrace planes show finer scale curved terrace edges (see below).

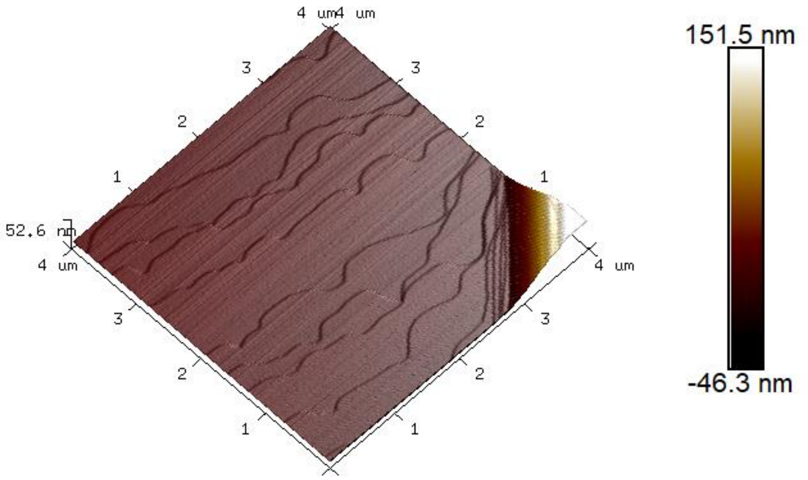

Figure S9. A higher magnification 3 x 3 μm AFM measurement of the Nle.HBr crystal surface showing discreet curved terraces. Note the larger scale straight terrace edge co-existing at the right corner of image.

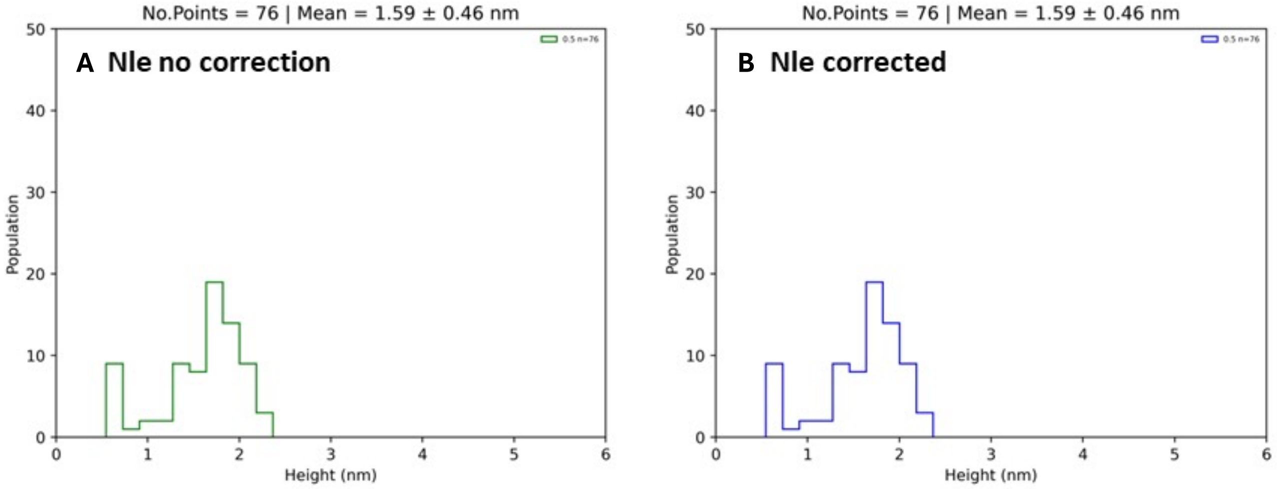

Figure S10. A) Histogram of IFC terrace step heights measured for the Nle.HBr sample, based on the AFM topography image shown in Figure S9. The data is compiled from step heights identified from line profiles shown in Figure S11. B) Recalculated step heights obtained after correcting for an artefact induced by routine AFM topography data processing. See further explanation associated with Figure S11 and the last section of the Experimental on “AFM Step Height Estimation”.

II.4 Characterization. The mean and standard deviations are conserved before and after the correction.

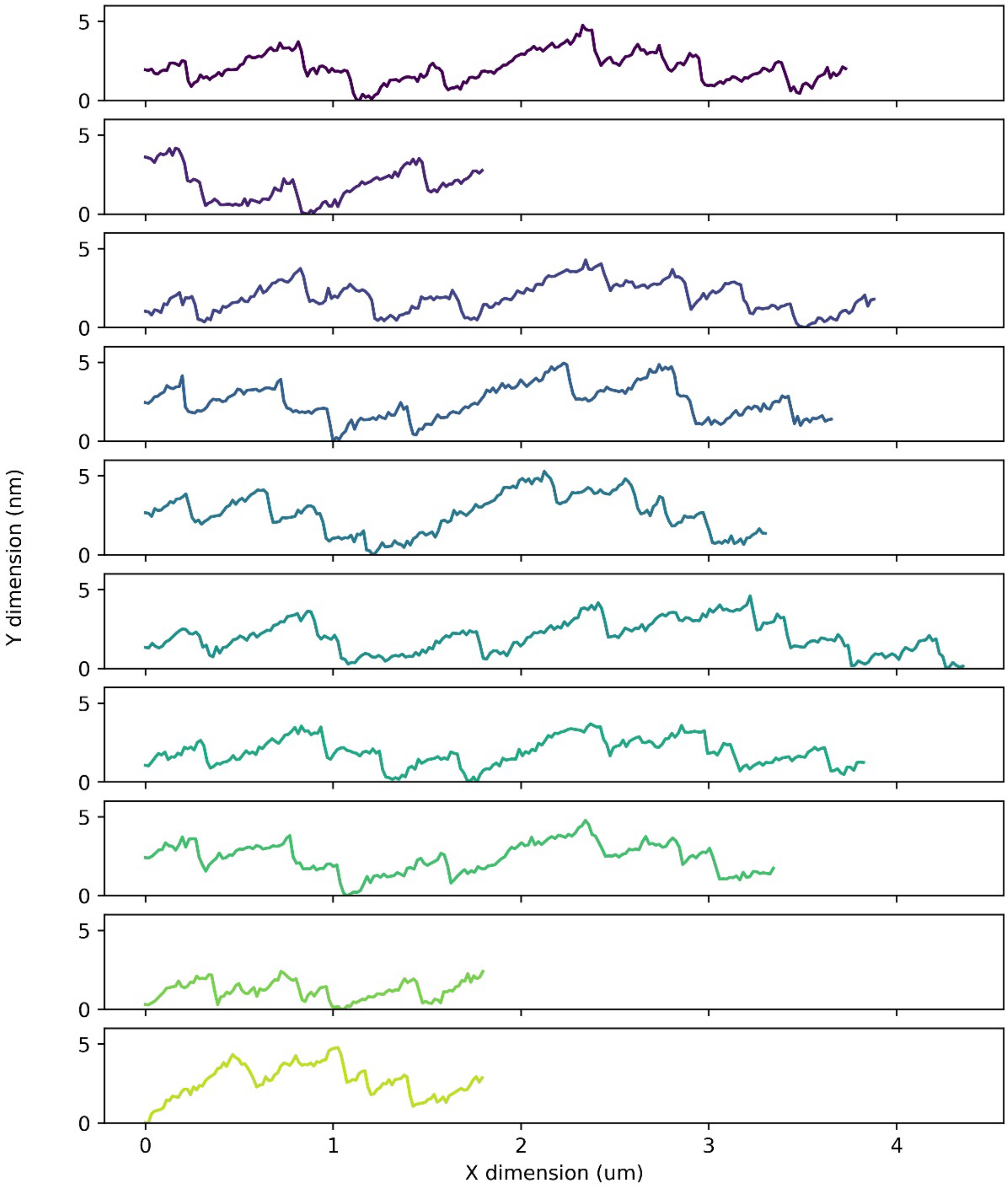

Figure S11. 10 line profiles measured on an AFM image taken for Nle.HBr (Figure S9).

The line profiles all exhibit a sawtooth pattern. This is an artefact from routine line flattening applied to the topography data, to remove small mismatches in height positions as the AFM tip scans one line of the surface after another. While the procedure was successful in removing the line-by-line discrepancies and revealed the terrace structure (Figure S8), the procedure assumed that the overall surface topography was flat. However, the IFC terrace structure meant that the imaged area had a higher height at one end of the image than the other, and line flattening has removed this overall topography. Instead, the originally flat terraces appeared as longer upward sloping segments and the terrace steps appeared as short slopes dropping in height, hence a sawtooth pattern. We have developed an algorithm to correct this artefact (see “AFM step height” in section II.2 Characterization) and the sawtooth pattern is visually diminished if the line plots were simply rotated slightly clockwise. An 11th corrected line profile is also shown in Figure 1D in the main text. In terms of calculating step heights, this geometric distortion had only a small practical effect

since the line flatterning was a simple height translation and the slope artefact was mostly minor. Therefore the step height calculated before and after the correction wre virtually identical (Figure S10).

I.3. Crystal Morphology Characterization by Bright Field Optical Microscopy of Nab, Nf, Nfe IFC Crystals and Nl non-IFC Crystals

Representative optical microscopy images of crystal surfaces obtained for 3 independent replicates of Nab.HBr IFC formation experiments.

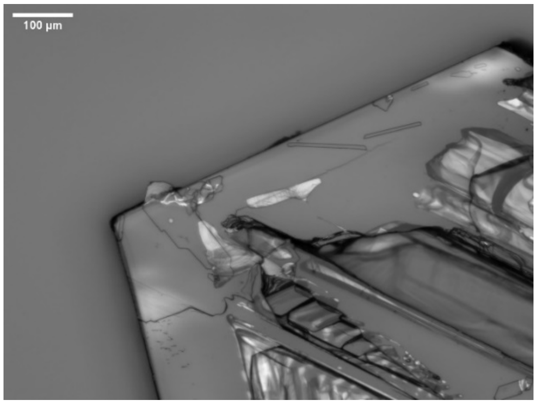

Figure S12. Nab.HBr sheet surface – experiment 1.

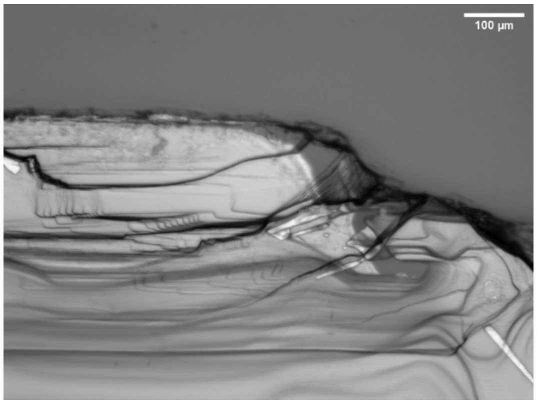

Figure S13. Nab.HBr sheet surface – experiment 2.

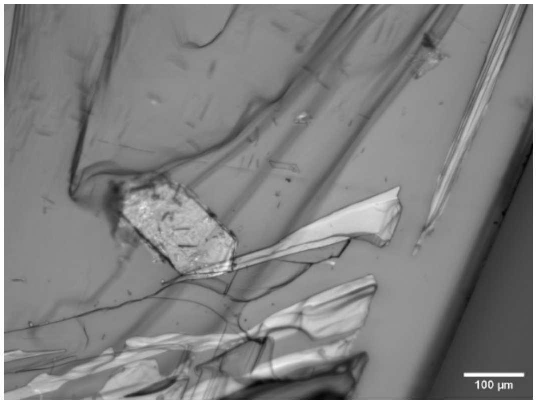

Figure S14. Nab.HBr sheet surface – experiment 3.

Representative optical microscopy images of crystal surfaces obtained for 3 independent replicates of Nf.HBr IFC formation experiments.

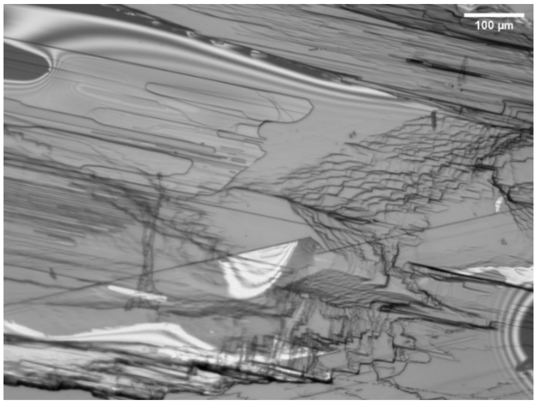

Figure S15. Nf.HBr sheet surface – experiment 1.

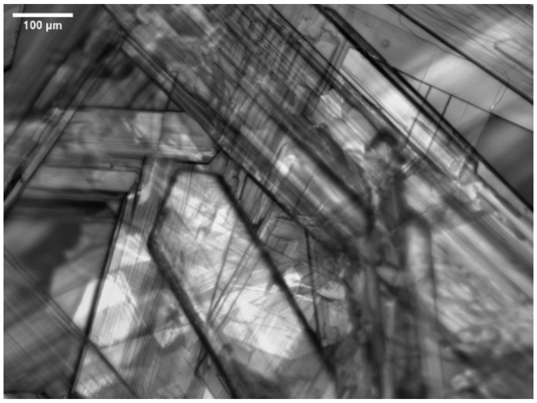

Figure S16. Nf.HBr sheet surface – experiment 2.

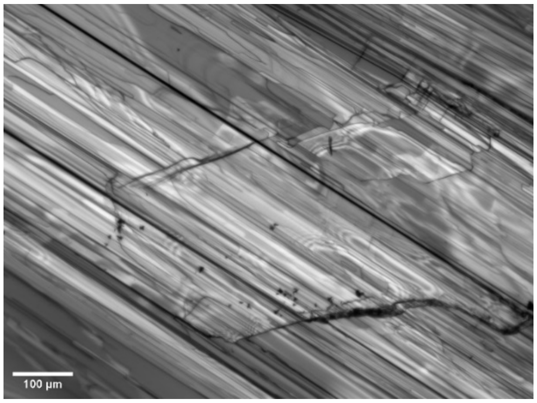

Figure S17. Nf.HBr sheet surface – experiment 3.

Representative optical microscopy images of crystal surfaces obtained for 3 independent replicates of Nfe.HBr IFC formation experiments.

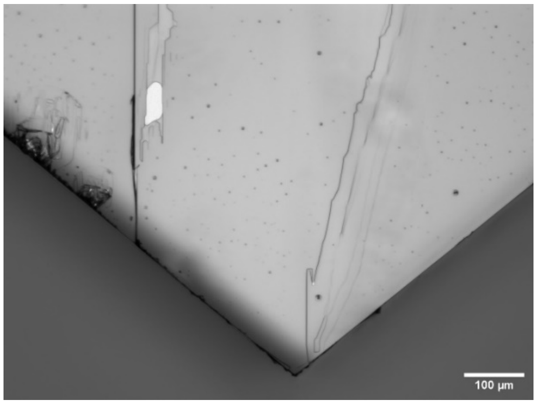

Figure S18. Nfe.HBr sheet surface – experiment 1.

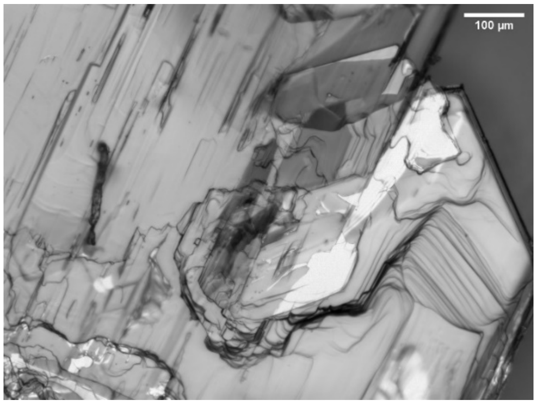

Figure S19. Nfe.HBr sheet surface – experiment 2.

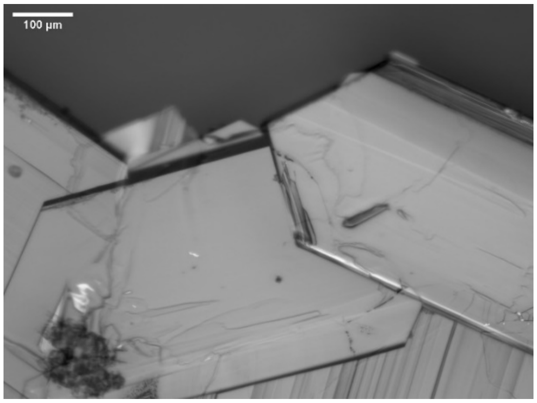

Figure S20. Nfe.HBr sheet surface – experiment 3.

Representative optical microscopy images of crystal surfaces obtained for 3 independent replicates of Nl.HBr crystallization experiments.

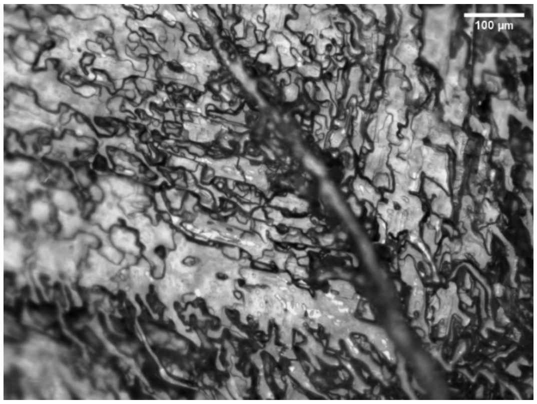

Figure S21. Nl.HBr crystal.

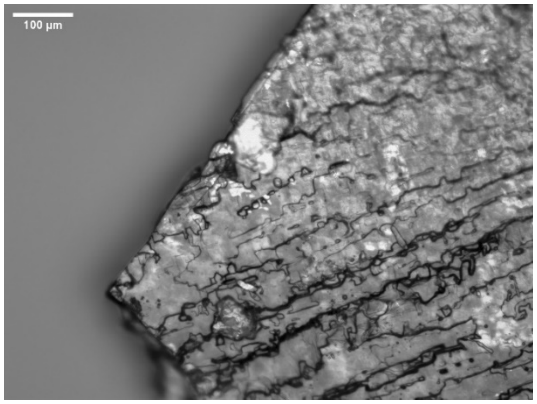

Figure S22. Nl.HBr crystal.

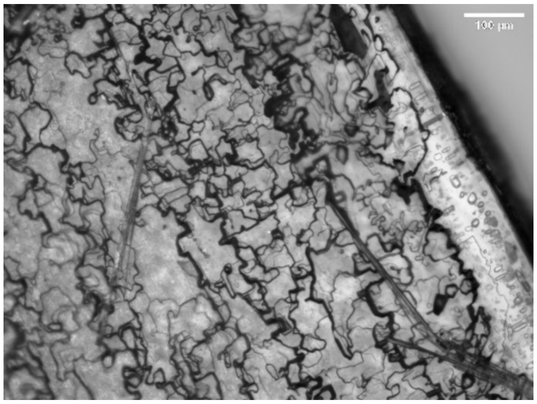

Figure S23. Nl.HBr crystal.

I.4. AFM Topography Measurements of Nab IFC Crystal and Nl Bulk Crystal

AFM Topography Imaging of Nab.Hbr Samples

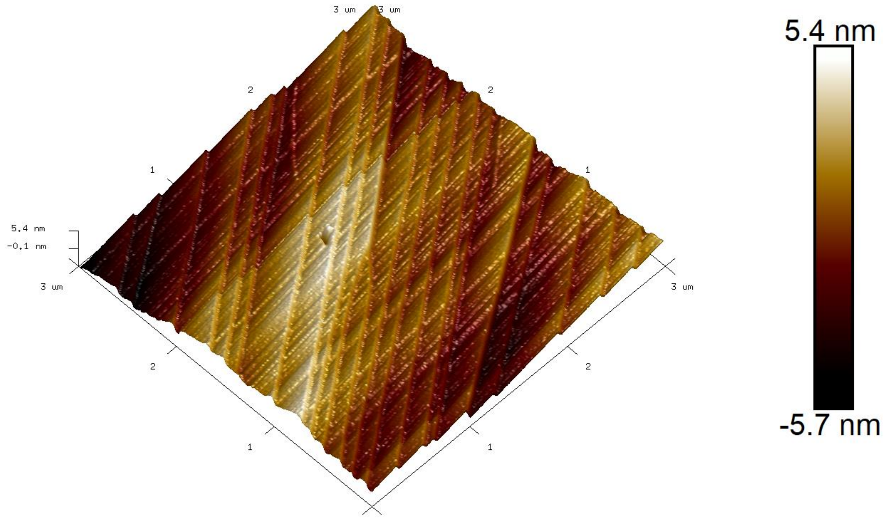

Figure S24. Representative 3 x 3 μm AFM image of Nab.Hbr IFC crystal surface showing terraced layers with well defined edges.

AFM Step Height Characterization for the Nab.Hbr IFC Sample Surface

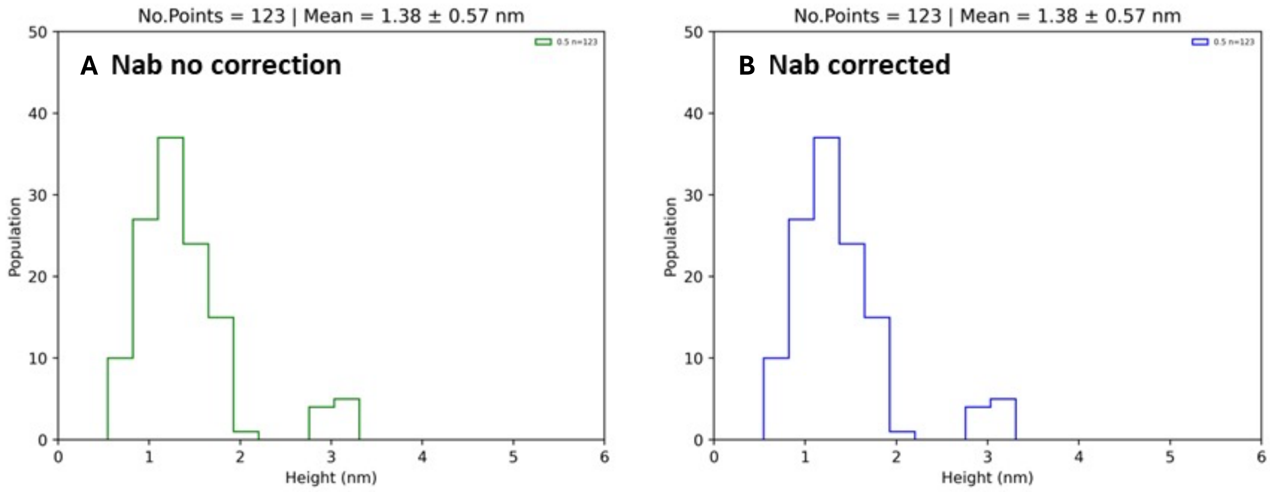

Figure S25. A) Histogram of IFC terrace step heights measured for the Nab.HBr sample, based on the AFM topography image shown in Figure S24. The data is compiled from step heights identified from line profiles shown in Figure S26. B) Recalculated step heights obtained after correcting for an artefact induced by routine AFM topography data processing. See further explanation associated with Figure S11 and the “AFM Step Height Estimation” section in II.4 Characterization. The mean and standard deviations are conserved before and after the correction.

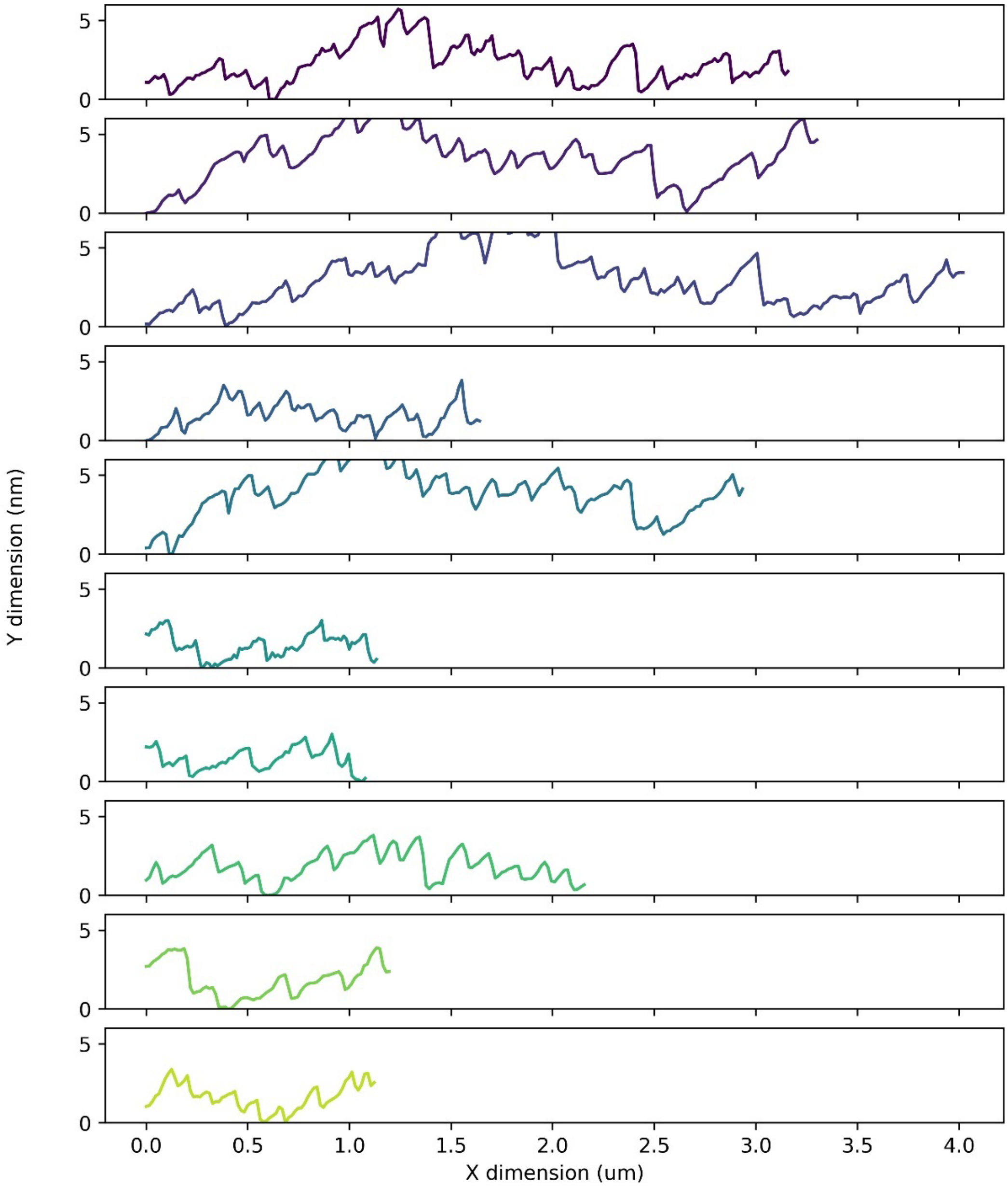

Figure S26. 10 line profiles measured on an AFM image taken for Nab.HBr (Figure S24). scan measurements taken for Nab.HBr, illustrating clear steps.

The line profiles all exhibit a sawtooth pattern but this is an artefact from routine AFM topography data processing. See Figure S11 and “AFM Step Height Estimation” in section II.4 Characterization for further explanation.

AFM Topography Imaging of Nl.Hbr Sample

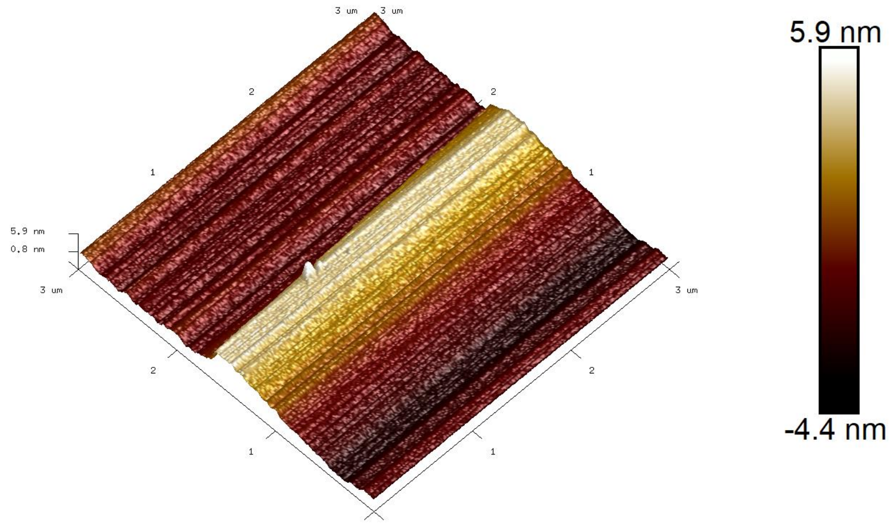

Figure S27. 3 x 3 µm AFM measurement of a Nl.HBr bulk crystal surface showing a lack of well-defined structure, consistent with optical microscopy observations (See S19).

I.5. ATR-FTIR Measurements

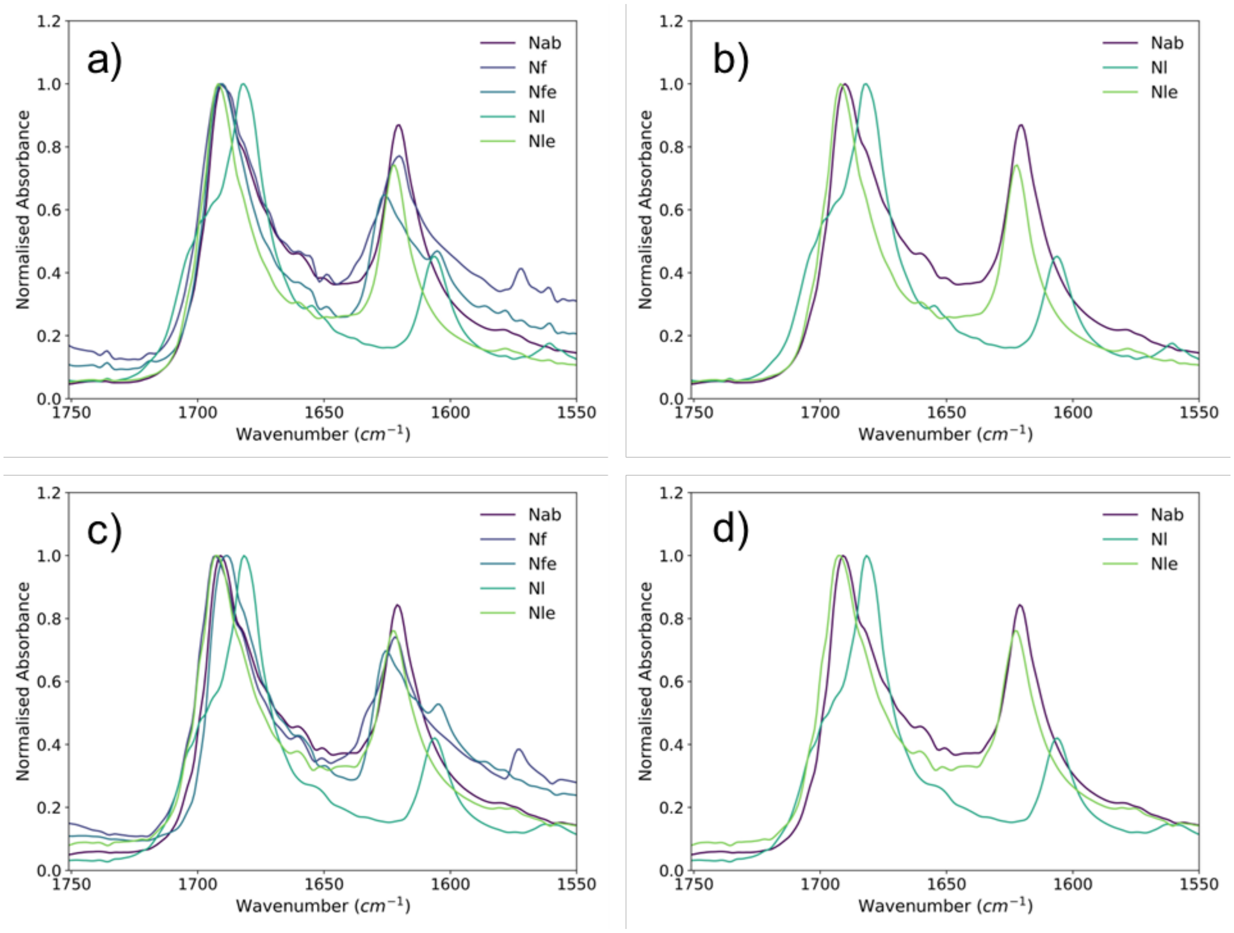

Figure S28. a) Normalized ATR-FTIR measurements for a second batch of crystals formed from all five peptoid amide monomers included in the study (first set of results shown in Figure 2 of the main text). b) Subset of the data showing only the results for aliphatic residues—both Nle and Nab formed IFC structures and Nl did not. c) Normalized ATR-FTIR measurements for a *third* batch of crystals formed from the five peptoid amide monomers. d) Corresponding subset of the data showing only the results for aliphatic residues Nab, Nl, and Nle. All batches of Nl.HBr show a different pattern of amide I and II shifts (shoulder instead of peak at ~1690 cm^{-1} and peak at ~1605 cm^{-1} instead of ~1620 cm^{-1}) compared to the rest of the peptoids that underwent IFC.

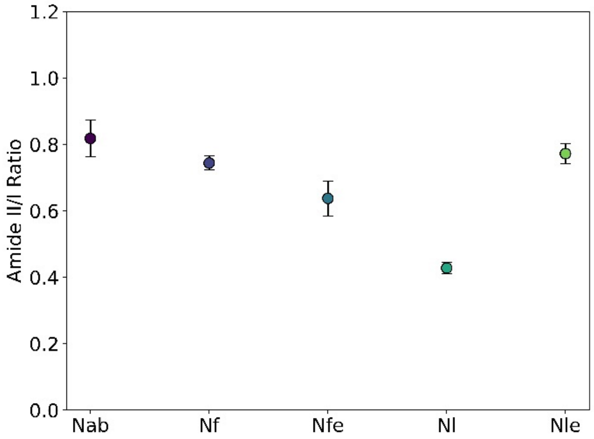

Figure S29. Amide II/I absorbance ratios averaged across three set of experiments for all peptoid measurements (original data shown in Figure 2 in the main text and in Figure S28 above). Clearly, the amide II/I ratio is substantially reduced for the Nl bulk crystals (~0.4) compared with all other species (0.65~0.82). The error bars show ±1 SD.

I.6. Hydrophobicity Comparisons of Aliphatic Peptoid Amide Monomers

Solvent Compositions for IFC Formation from Peptoid Amide Monomers

Table S1. Specific solvent compositions for comparative crystallization study. See Experimental section on crystal preparation (II.3) for details on methodology.

	Experiment 1		Experiment 2		Experiment 3		
**Monomer Salt**	**Mass (mg)**	**methanol (mL)**	**Mass (mg)**	**methanol (mL)**	**Mass (mg)**	**methanol (mL)**	**Average (mL)**
**Nab**	30.3	0.410	29.7	0.420	30.4	0.420	0.417
**Nl**	30.1	0.220	29.7	0.230	30.0	0.230	0.227
**Nle**	30.2	1.000	30.2	1.000	30.2	1.000	1.000
**Nf**	29.8	0.830	30.1	0.850	30.3	0.890	0.857
**Nfe**	30.5	1.140	30.3	1.150	30.5	1.200	1.163

RP-HPLC and Solubility Experiments

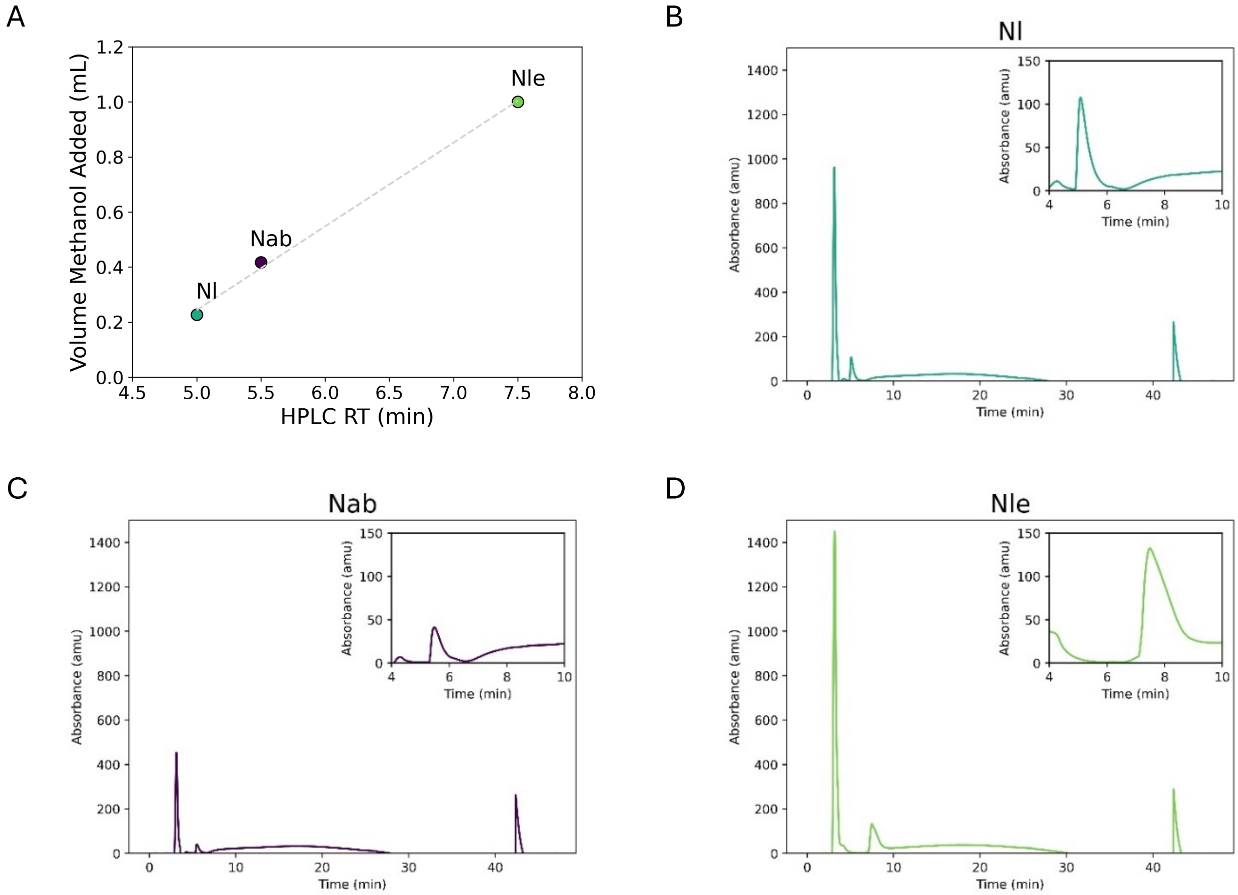

Figure S30. A) Plot of RP-HPLC retention time vs. the volume of methanol added to acetonitrile (ACN) in order to precipitate the dissolved aliphatic peptoid amide monomers (see data in Table S1). B, C, D) Original RP-HPLC chromatograms of Nl, Nab, and Nle monomers, respectively, with absorbances measured at 214 nm.

In the HPLC chromatograms, longer elution times indicated higher hydrophobicity since a 5% to 95% ACN:water gradient was used (see Experimental: Characterization). Other than the unretained TFA peak at 3 min and a wash peak at 43 min that are common to all elutions, the insets show that the purified Nl, Nab, and Nle all exhibited single elution peaks at ~5 min, ~5.5 min, and ~7.5 min, respectively. These values are plotted as the x-axis in panel A above. Also, the affinity for the less polar ACN is higher for more hydrophobic monomers such that more methanol must be added to reduce the solubility in the mixture sufficiently to enable precipitation. This is indicated by the y-axis values in panel A above.

LogP Calculation

Structures of all candidate IFC molecules were built using Gaussview[1] in the neutral charge state, and LogP was calculated using the RDKit[2] implementation of Wildman and Crippens atomic contribution approach for LogP calculation.[3]

Table S2. Calculated LogP Values for candidate IFC molecules

Monomer Salt	Mass (mg)
**V-NH_2**	-0.7532
**L-NH_2**	-0.3631
**Nl**	-0.4909
**Nab**	-0.3468
**Nle**	-0.1008
**Nf**	0.4334
**Nfe**	0.8235

I.7. Amino Acid Amide IFC Crystal Morphologies Characterized by Optical Microscopy

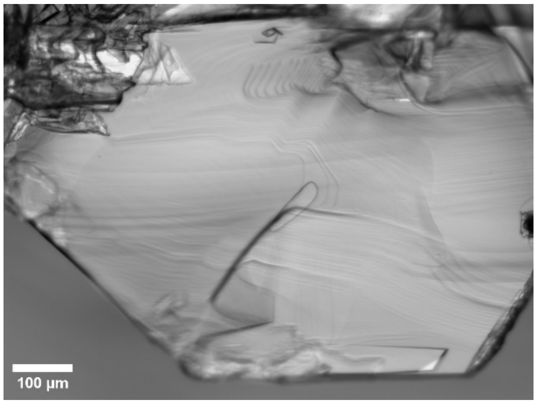

Figure S31. IFC formed by L-NH_2.HCl showing clearly defined terraced layers as found in the peptoid amide IFCs suggesting a shared growth mechanism.

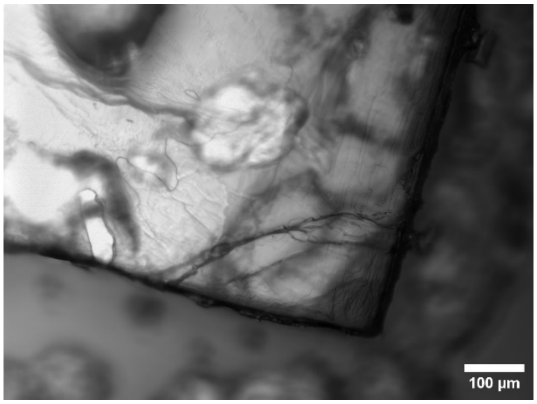

Figure S32. IFC formed by V-NH_2.HCl also showing terraced layers though the crystals are less well defined than their leucine-amide counterparts.

I.8. $N_{ter} - C_\alpha - C_\beta - C_\gamma$ Torsion Scans

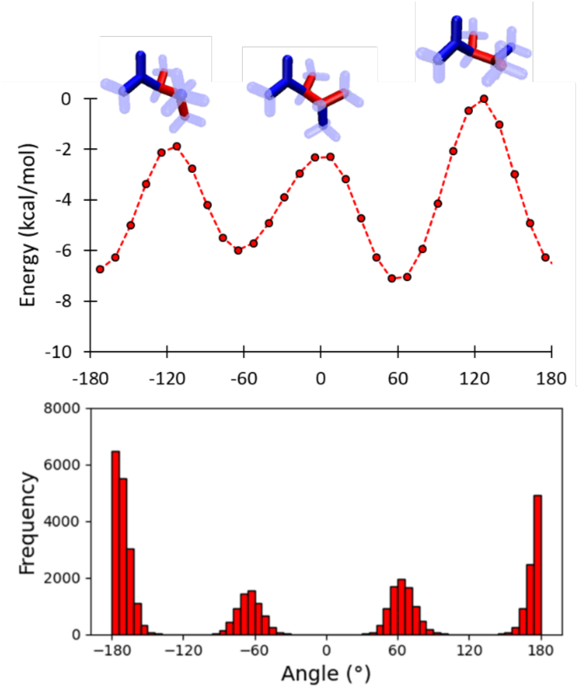

Figure S33. N_{ter}–C_α–C_β–C_γ torsional scan for V-NH_2 at MP2/6-31G(d) level of theory and sampling frequency extracted from MD simulations. Interestingly, this shares similarities with Nl, suggesting that this profile is characteristic of a backbone-adjacent *ipso* group. MD sampling shows good agreement with QM energy minima obtained.

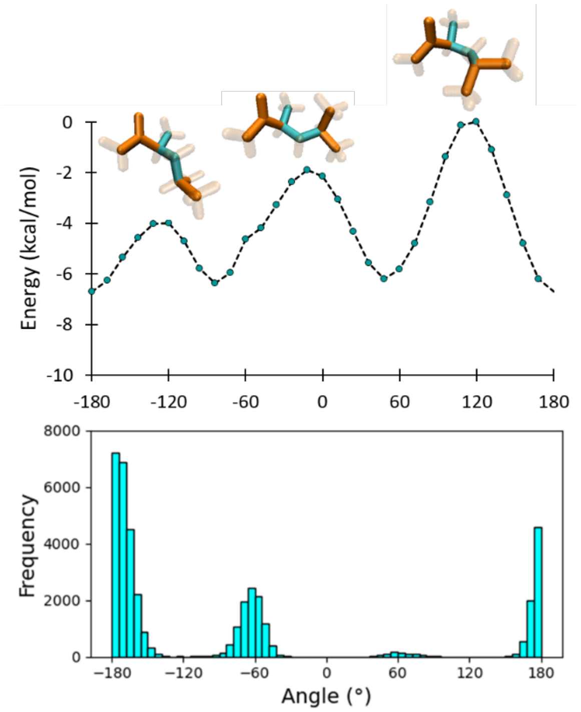

Figure S34. N_{ter}–C_α–C_β–C_γ torsional scan for L-NH_2 at MP2/6-31G(d) level of theory and sampling frequency extracted from MD simulations which are in good agreement. Notably the functional form of the torsion is distinct from that of the studied peptoid monomers and V-NH_2 while the IFC phenomena is conserved. This result suggests that for this amino acetimide salt sidechain confromation and IFC packing are decoupled.

I.9. Single Crystal X-Ray Crystallography Results

Selected crystallographic and refinement parameters are given below in Table S3 for Nle.HBr and in Table S4 for Nab.HBr. Full information in cif format has been deposited with the CCDC as reference number 2376002 and 2340476 for Nle.HBr and Nab.HBr, respectively.

Figure S35 further below shows the hydrogen bonding network in the two crystals.

See the X-Ray Crystallography section II.4 Characterization in the Experimental for dexcription of the measurements and structure determination.

Table S3. Selected Crystallographic and Refinement Parameters for Nle.HBr.

**Compound**	**Nle.HBr**
**Formula**	$C_7H_{17}BrN_2O$
**Form. Wt.**	225.13
**Crystal system**	Triclinic
**Space group**	P-1
**Temp. (K)**	200(2)
**a (Å)**	4.8029(3)
**b (Å)**	5.7754(3)
**c (Å)**	19.2543(9)
**α (°)**	93.312(4)
**β (°)**	94.658(4)
**γ (°)**	90.029(4)
**Volume ($Å^3$)**	531.43(5)
**Z**	2
**Collected Reflections**	7237
**Observed Reflections**	7001
**2Θmax (°)**	142.07
**No. Parameters**	109
**S**	1.182
**R [on *F*, obs refs only]**	0.0758
**ωR [on F^2, all data]**	0.2029
**Twin scale factor**	0.373(6)

Table S4. Selected Crystallographic and Refinement Parameters for Nab.HBr.

**Compound**	**Nab.HBr**
**Formula**	$C_6H_{15}BrN_2O$
**Form. Wt.**	211.11
**Crystal system**	Triclinic
**Space group**	P-1
**Temp. (K)**	100(2)
**a (Å)**	4.7441(4)
**b (Å)**	5.8024(4)
**c (Å)**	17.4186(10)
**α (°)**	89.967(6)
**β (°)**	89.204(6)
**γ (°)**	89.987(6)
**Volume (Å^3)**	479.44(6)
**Z**	2
**Collected Reflections**	6525
**Independent Reflections**	1769
**R_{int}**	0.0445
**Observed Reflections**	1658
**2Θmax (°)**	143.32
**No. Parameters**	143
**S**	1.102
**R [on *F*, obs refs only]**	0.0582
**ωR [on F^2, all data]**	0.1720

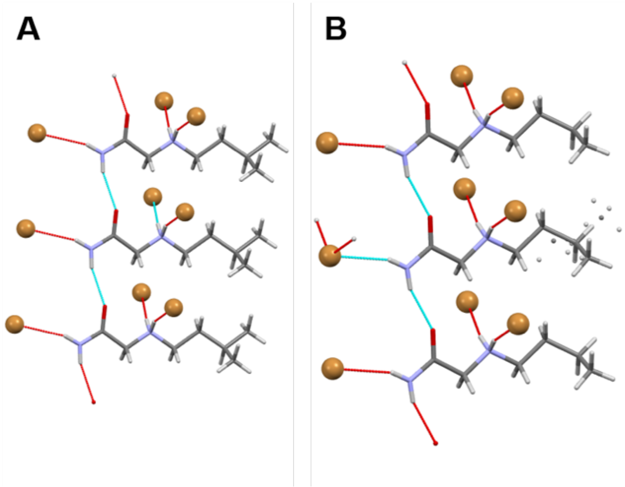

Figure S35. Single X-ray diffraction structures of Nle.HBr (A) and Nab.HBr (B). The presence of intermolecular hydrogen bonding via the N-termini (blue) and 2:1 ionic coordination between anions and protonated amine/amide termini (red) show that both modes of intermolecular noncovalent interactions are important in the formation of IFC crystals. Furthermore, the extension of the sidechain with χ_2 ~ ± 180° supports the observed sampling in MD simulations for these species, as well as the energy minima in QM torsion scans (see Figure 3 in the main text).

I.10. Comparison of the Morphologies of Nle Crystals Formed with Chloride and Bromide Salts, as Characterized by Optical Microscopy

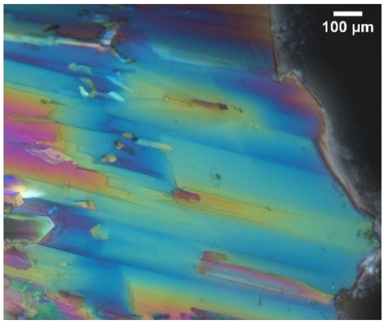

Figure S36. Nle.HCl with polarised light

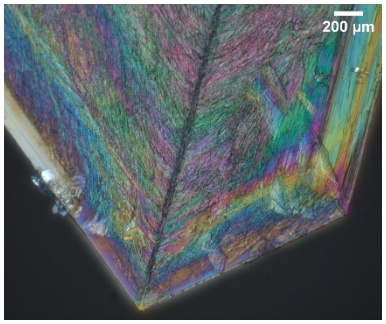

Figure S37. Nle.HBr with polarised light

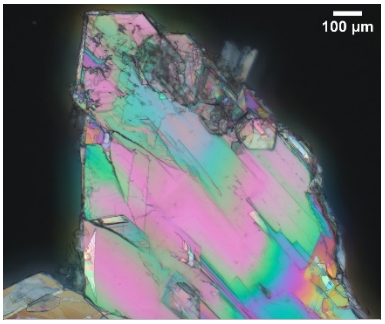

Figure S36: Nle.HCl with polarised light

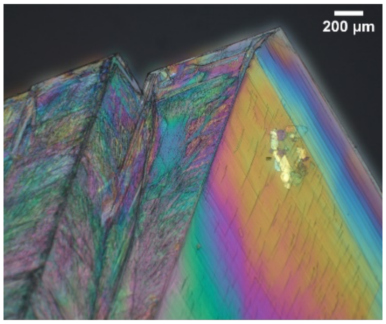

Figure S38. Nle.HBr with polarised light

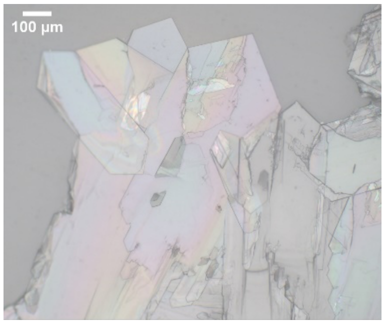

Figure S39. Nle.HCl with polarised light

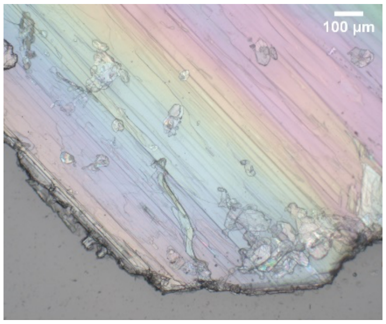

Figure S40. Nle.HBr with polarised light

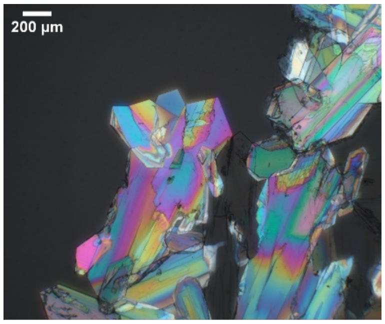

Figure S41. Nle.HCl with polarised light

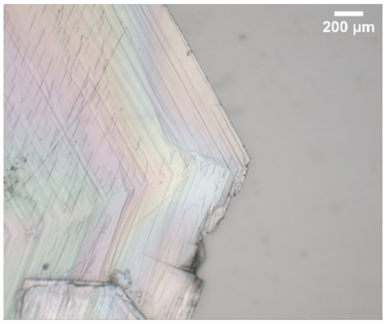

Figure S42. Nle.HBr with polarised light

I.11. Molecular Dynamics Solution Measurements

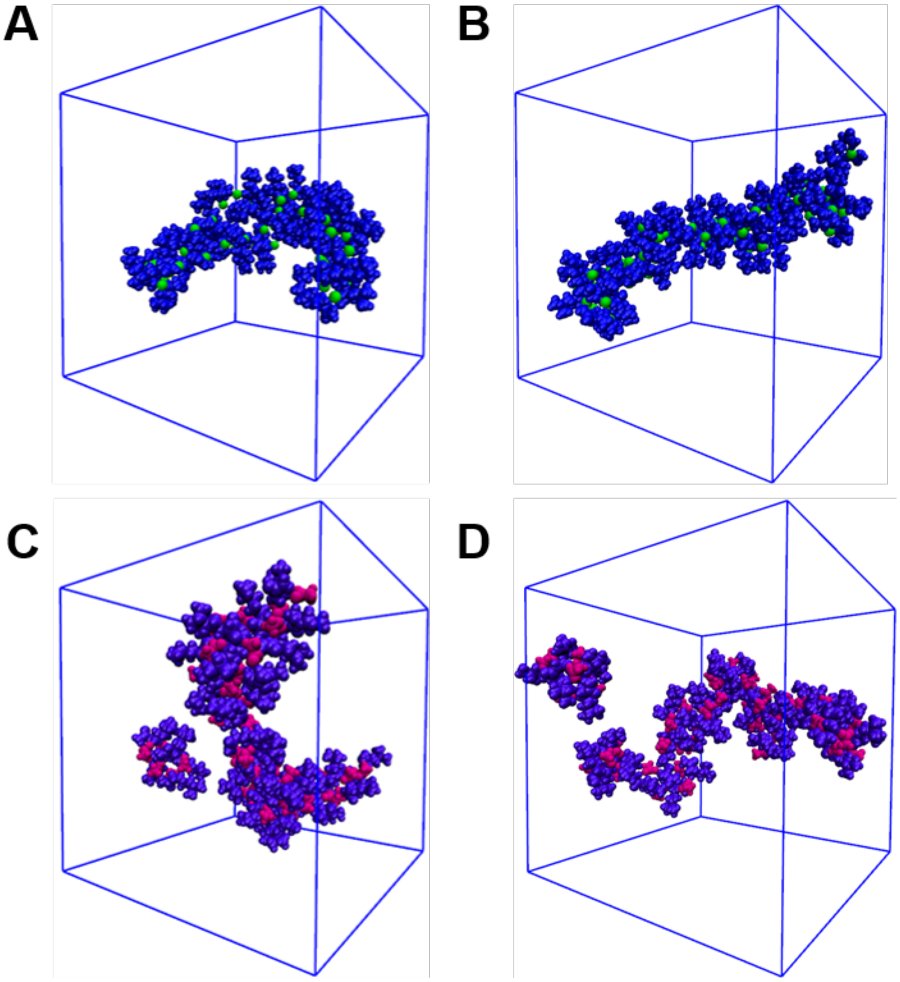

Figure S43. Final structures from two separate 100 ns MD simulations of Nle.HCl (A and B) and Nle.H-acetate (C and D) in ACN (snapshots obtained after centering and clustering). Nle molecules are those in blue, while green and pink beads correspond to chloride and acetate anions respectively.

The snapshots show that the Nle.HCl structure is continuous and more compact in comparison with the disjointed aggregates obtained for Nle.H-acetate, which agrees with the hypothesis that acetate anions have higher solvent affinity and thus reduced thermodynamic drive to migrate from solution to interfaces and they are therefore unsuitable to template IFC growth.

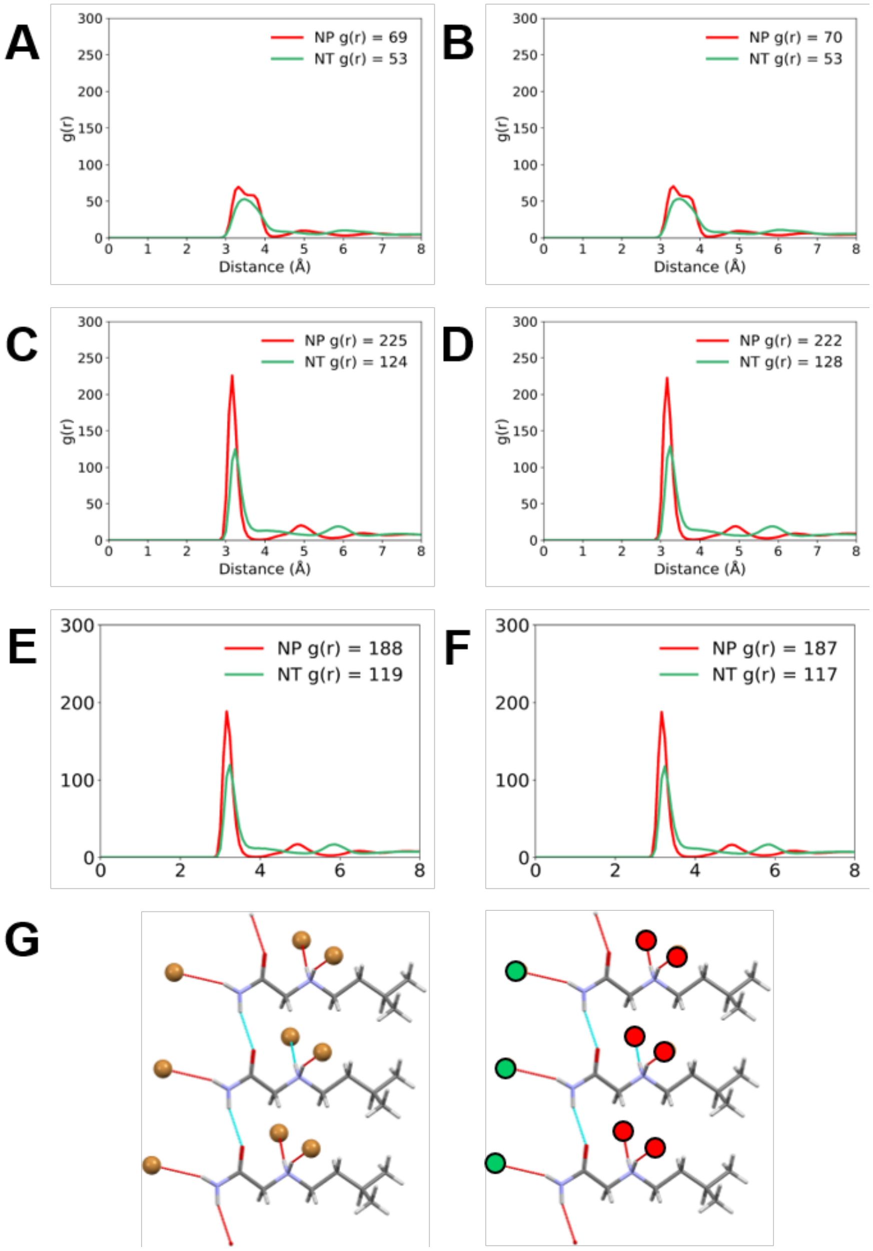

Figure S44. Duplicate RDFs for Nle.H-acetate (A and B), Nle.HCl (C and D), and Nab.HCl (E and F). (G) Illustration of coordination centers about which RDFs are calculated for the Nle.HBr structure.

The distinctly different RDFs between Nle in the presence of chloride and acetate anions supports the hypothesis that ion type is an important determinant of IFC propensity, where the acetate case does not form an IFC structure. For Nl, Nle and Nab, the ratio between the numbers of chloride ions coordinating with the N-terminus and with the amidated C-terminus are ~1:1, ~2:1 and ~1.6:1, respectively. The ~2:1 coordination for Nle corresponds directly to the measured crystal structure. While exact agreement is not observed for Nab.HCl, the ~1.6:1 ratio obtained from those simulations was larger than unity and would be sufficient to start inducing a continuous structure. The ~1:1 ratio of the Nle.H-acetate system would be less favorable for bridging multiple monomers, which is consistent with experiments showing no IFC formation for the Nle.H-acetate. To ensure an equal number of sites were used, we calculated the acetate RDF using the carboxylate carbon in the acetate anion.

Recently, we reported a forcefield natively developed for simulating peptoid molecules.[13] However, this was parameterized for an aqueous environment, and it is not strictly transferable to the acetonitrile environment of this study. Nonetheless, when applied, we were able to qualitatively reproduce peptoid-amide salt self-assembly, although the 2:1 ratio (see Section I9 and Figures S43 and 44) of anion contacts between the N-terminal and C-terminal amide centers observed in the Nle.HCl and Nab.HCl crystal structures was not obviously recovered. Analysis of the radial distribution function (RDF) using this recent peptoid model yielded two peaks for the N-terminal center (Figure S45), the first corresponding to coordination via $NH_2(+)$ group and the second at 4.2 Å (as indicated by black arrow) corresponding to coordination from the backbone $C_\alpha H_2$ unit which is quite electrophilic in our model.

In the parameterization of partial charges for the peptoid N-terminus, we targeted the reproduction of water binding energies from multiple complexes, and this is a markedly different electrostatic environment from the current IFC system (*i.e.*, ionic salt coordination in acetonitrile media). As such the lack of transferability is not entirely surprising. However, we find this result to be of interest as it highlights the electrophilic nature of the $C_\alpha H_2$ unit within the a peptoid type backbone structure. Experimentally we can observe this *via* the NMR characterization done for the bromide salts of Nl, Nab and Nle, for which the protons at the C_α position are considerably more deshielded than the C_β position with shifts of ~3.85 and ~ 3 ppm respectively (Figures S47, S49 and S50).

In the broader field of peptoid research, we note that crystal structures of cyclic peptoids have previously been found to form stable CH – O carbonyl hydrogen bonds, indicating that the electrophilic nature of the $C_\alpha H_2$ unit is sufficient to make this unit a proton donor.[14] It is tempting to speculate that the polarizable nature of the backbone $C_\alpha H_2$ unit might also be linked to the differences in amide bond *cis/trans* isomerism which are known to be solvent dependent for certain peptoid monomers,[15] though further investigations are required to better establish this connection.

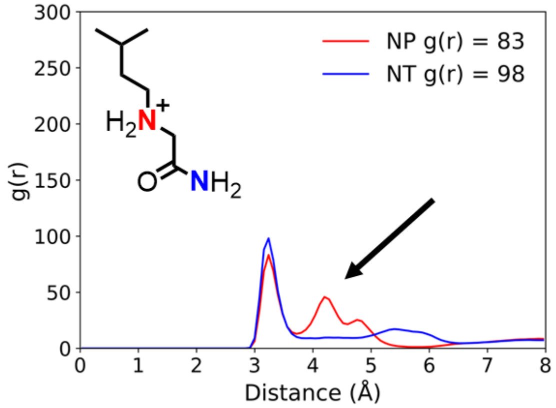

Figure S45. Radial distribution functions (RDFs) of chloride anion and N-centers in Nle peptoid monomer using our recently parameterized peptoid N-terminus partial charge model. A split coordination shell was observed for the N-terminal $NH_2(+)$ group which arises due to the electrophilic character of the adjacent $C_\alpha H_2$ unit, as obtained by fitting partial charges towards the reproduction of water binding energies in multiple interaction complexes. This result is of interest as it highlights the polarized nature of the $C_\alpha H_2$ unit in peptoid backbones, which is also observed *via* NMR measurements in D_2O solvent.

I.12. Molecular Dynamics Simulations at an Acetonitrile:Vacuum Interface

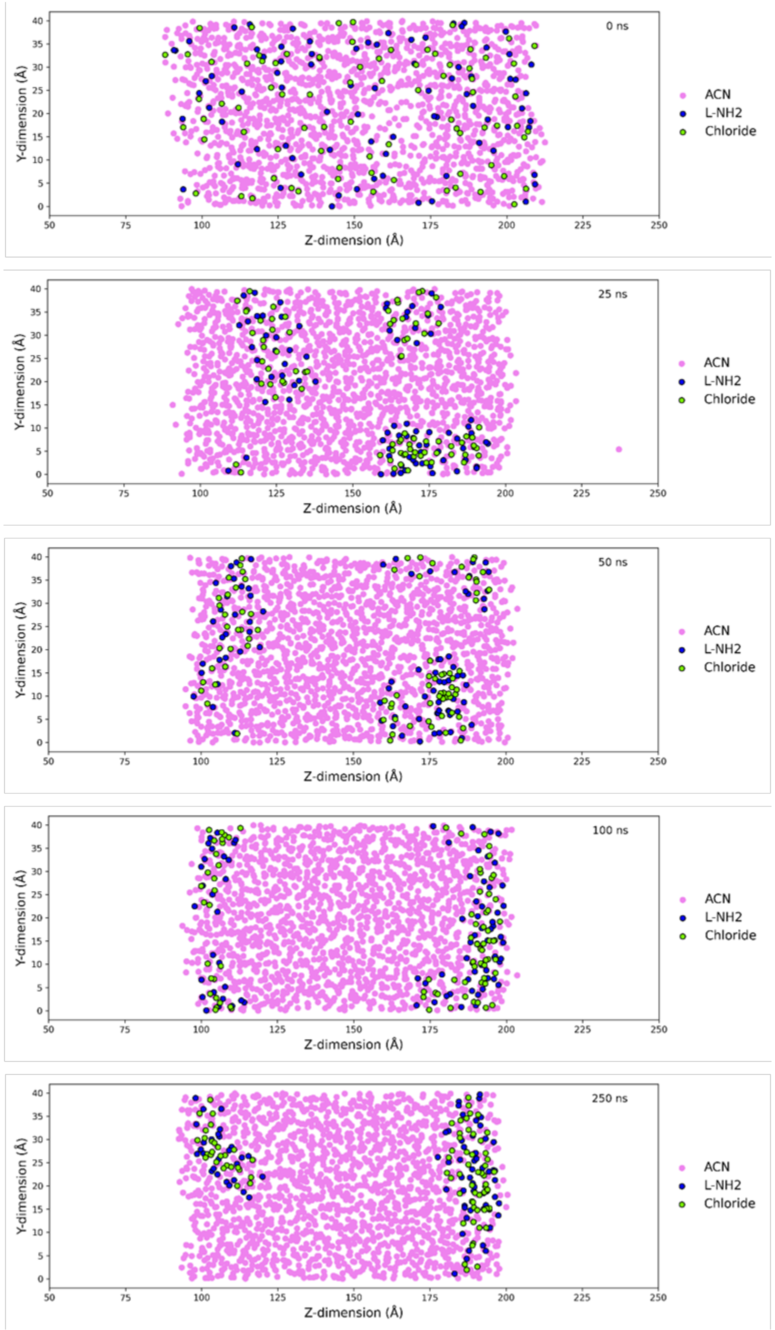

Figure S46. Example y/z perspectives of the acetonitrile:vacuum interface simulated for 250 ns in the NVT ensemble for L-NH_2.HCl, where constituent entities are represented by their collective center of mass (COM). From top to bottom, snapshots at increasing 50 ns intervals are shown.

Additional MD simulations were performed with an acetonitrile:vacuum interface, simulating the experimental air-liquid interface, for both L-NH_2.HCl and V-NH_2.HCl. These simulations were performed in the NVT ensemble, for 250ns, and in triplicate. Assembly in the solution phase was found in all simulations, and migration of these clusters to the vacuum phase boundary was also

observed, illustrating a possible mechanism for IFC formation. Figure S46 shows example snapshots of the L-NH_2.HCl system. In some replicates (not shown), assemblies were seen to pass back into the solution phase. We interpret this as highlighting the potential importance of solvent evaporation, and the concurrent changes in surface tension, which cannot be readily captured when using the NVT ensemble, but which could influence the partition of assemblies at the interface in real systems and play a role in driving IFC. At the liquid-liquid interface, there could be analogous effects of diffusion gradients across the interface.

II. EXPERIMENTAL (including Tables S5 and S6, and Figures S47 to S53)

II.1 Materials and Synthesis of Peptoid Amide Monomers

2-bromoacetamide (Sigma Aldrich) was dissolved in acetonitrile (0.28 M, Fischer Scientific) and to this, 1 equivalent of the desired amine was added with stirring (see Table S1 for suppliers). All residues formed a precipitate after 12 – 24 hrs. Purification by recrystallization was then done in ~ 2: 1 v/v acetonitrile: methanol (temperatures > 50°C). Crystalline material was isolated by filtration and washed with cold solvent, yields were modest, between 42 – 56 %, reflecting that the salts all have some solubility in the recrystallization solvent. Purity was confirmed by NMR (see Figure S47 through Figure S52). In some batches 1 equivalent N,N-Diisopropylethylamine (DIPEA, Alfa Aesar) was added to quench HBr. However, this did not have a significant impact on the resulting yield.

Table S5. Suppliers of amine starting materials

**Monomer**	**Supplier**
Isopentylamine	Alfa Aesar
Isobutylamine	Sigma Aldrich
Butylamine	Alfa Aesar
Phenylethylamine	Apollo Scientific
Benzylamine	Sigma Aldrich
H-Leu-NH_2.HCl (L-leucinamide hydrochloride)	Sigma Aldrich
H-Val-NH_2.HCl (valinamide hydrochloride)	Bachem

II.2 Crystal Preparation

IFC crystals were prepared by dissolving solid monomer material in a mixture of acetonitrile:methanol in varying proportions with heating above 50°C. Crystals of the same morphology were formed over the temperature range 50 – 80°C (see Table S5 for amino acid amide suppliers). After all material was dissolved, the solution was partially covered and left to cool. To assess how crystal morphology changed with sidechain functionality, 50 mg of each salt material was combined with 2 mL of acetonitrile and sufficient methanol for total dissolution with heating (see Table S3 for compositions). The solutions were covered to cool, then uncovered in a slightly open box for slow evaporation. The resultant morphologies formed were highly reproducible across triplicate independent experiments (see optical microscopy characterization images in Supplementary Figures sections I.2 and I.3).

Table S6. Yields for Nle neutral amine work up.

**Purification**	**Starting Material (mg)**	**Yield (%)**
1	280.0	31.4
2	249.5	84.1

II.3 Experiments to Screen Anion Type

To obtain the neutral amine, a desired mass of Nle.HBr (Table S3) was combined with NaOH solution (1M, 70 mL), followed by extraction using sequential washes with dichloromethane (1 x 70 mL) and chloroform (2 x 70 mL). After drying with anhydrous magnesium sulfate, solvent was removed

by rotary evaporation. The resultant oily liquid was transferred to a beaker with minimal DCM washes. Purification by recrystallization with hexane followed and the crystalline material was separated by filtration (see Table S2 for yields). Purity was confirmed by NMR (Figure S48). Equimolar equivalents of acetic, valeric, sulfuric and hydrochloric acid (duplicated) were added to 30 mg of the neutral amine. The solutions were then lyophilized after freezing and ~2:1 v/v acetonitrile:methanol solvent was added with heating until full dissolution of the material. This experiment was also repeated for Nle.HBr (50 mg). It was found that only the halide salts formed IFCs, and this was still the case after the vials were left open on the bench for 72 hrs. The structure of crystals formed by Nle.HCl had the same morphology as Nle.HBr (see optical microscopy characterization images Figure S36 through Figure S42).

II.4 Characterization

Nuclear Magnetic Resonance (NMR) Spectra for Purified Peptoid Amide Monomers
^{1}H NMR spectra were recorded on a Bruker Avance III 400 MHz spectrometer.

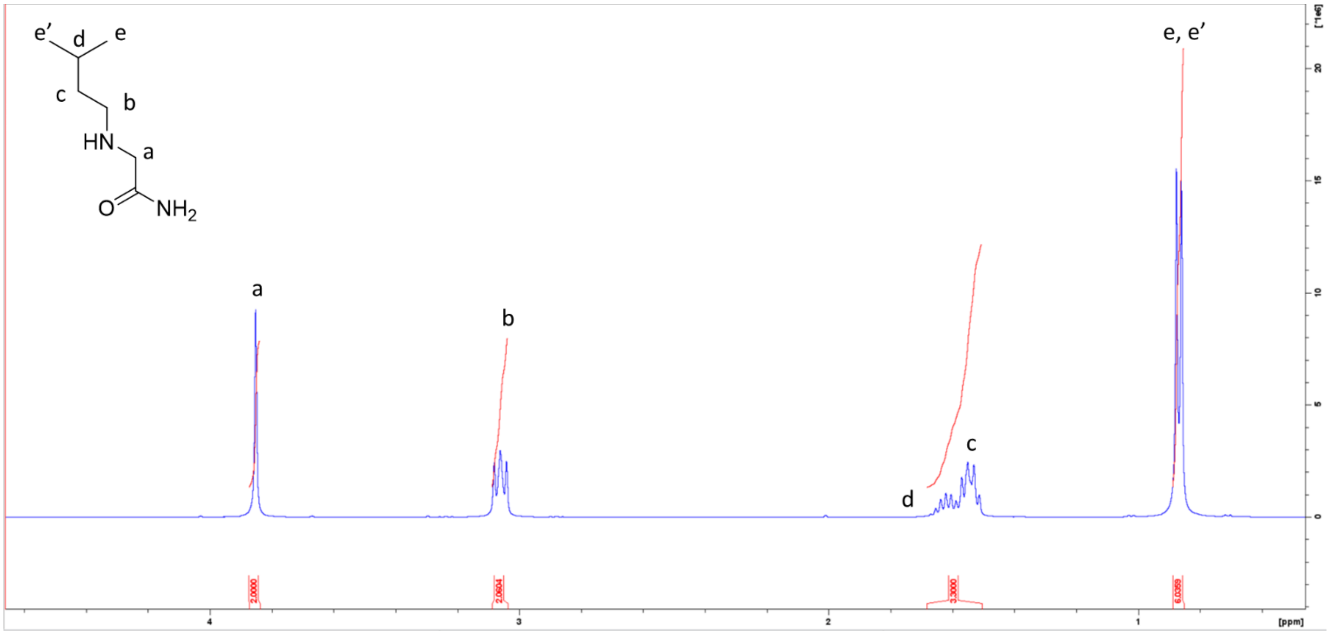

Figure S47. ^{1}H NMR of.Nle.HBr (400 MHz, D_2O) δ (ppm): 0.87 (d, 6H), 1.54 (q, 2H), 1.62 (nonet, 1H), 3.06 (t, 2H) and 3.85 (s, 2H)

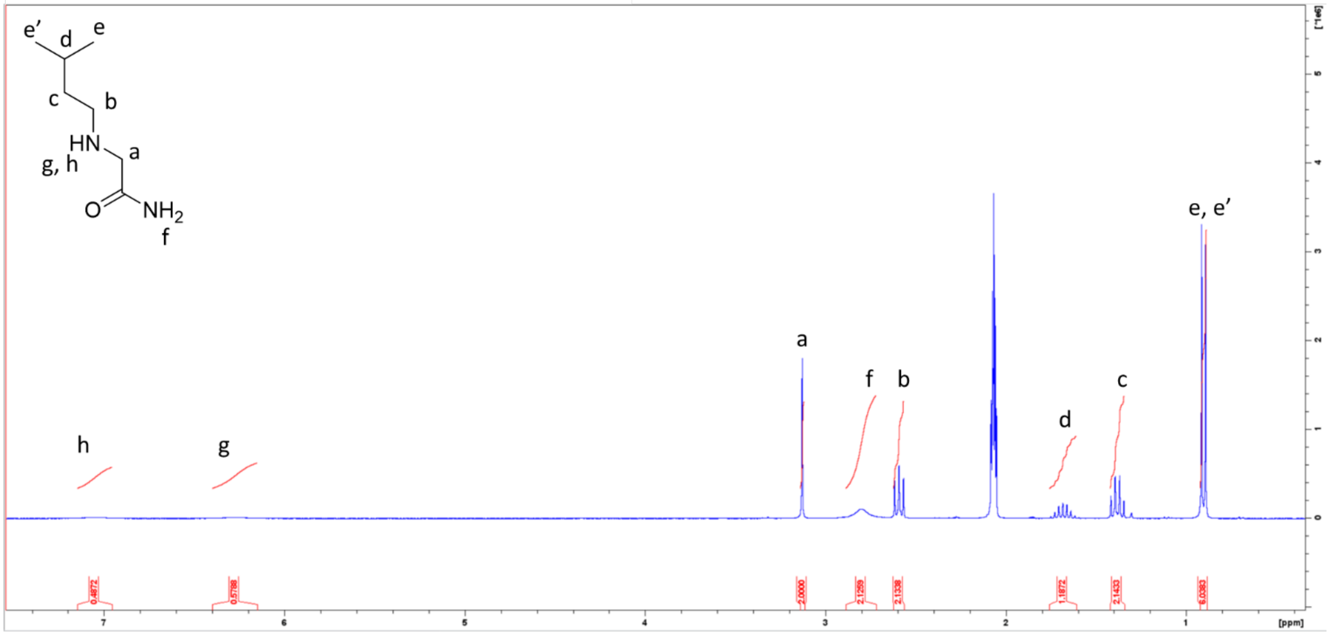

Figure S48. ^{1}H NMR of Nle neutral amine for salt screening experiments (400 MHz, d6-acetone) δ (ppm): 0.905 (d, 6H), 1.38 (q, 2H), 1.68 (nonet, 1H), 2.60 (t, 2H), 2.80 (s, 2H), 3.12 (s, 2H), 6.27 (s, 0.58) and 7.06 (s, 2H). (Note: peaks at g and h are interpreted as the N-terminal proton which is split between two conformations).

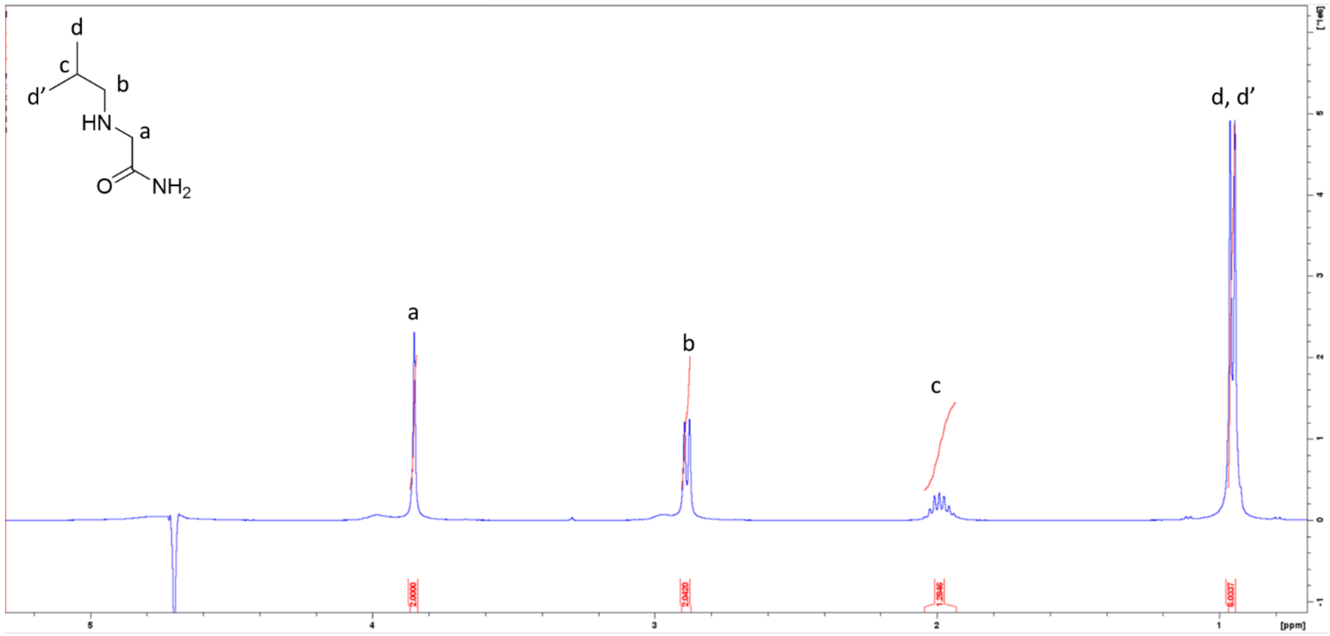

Figure S49. ^{1}H NMR of Nl.HBr (400 MHz, D_2O) δ (ppm): 0.95 (d, 6H), 1.99 (nonet, 1H), 2.89 (d, 2H) and 3.85 (s, 2H). (Note: inverted signal at 4.70 ppm is H_2O).

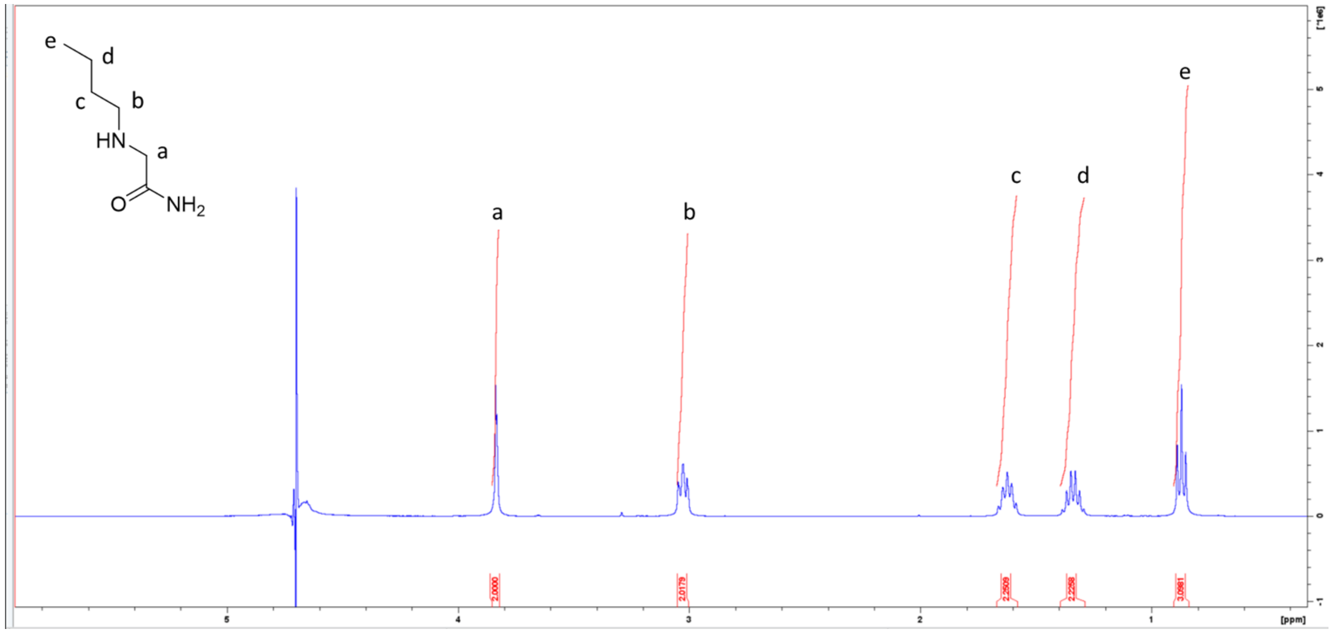

Figure S50. ^{1}H NMR of Nab.HBr (400 MHz, D_2O) δ (ppm): 0.87 (t, 6H), 1.34 (sextet, 2H), 1.63 (quintet, 2H), 3.02 (t, 2H) and 3.84 (s, 2H). (Note: 4.70 ppm shift is H_2O).

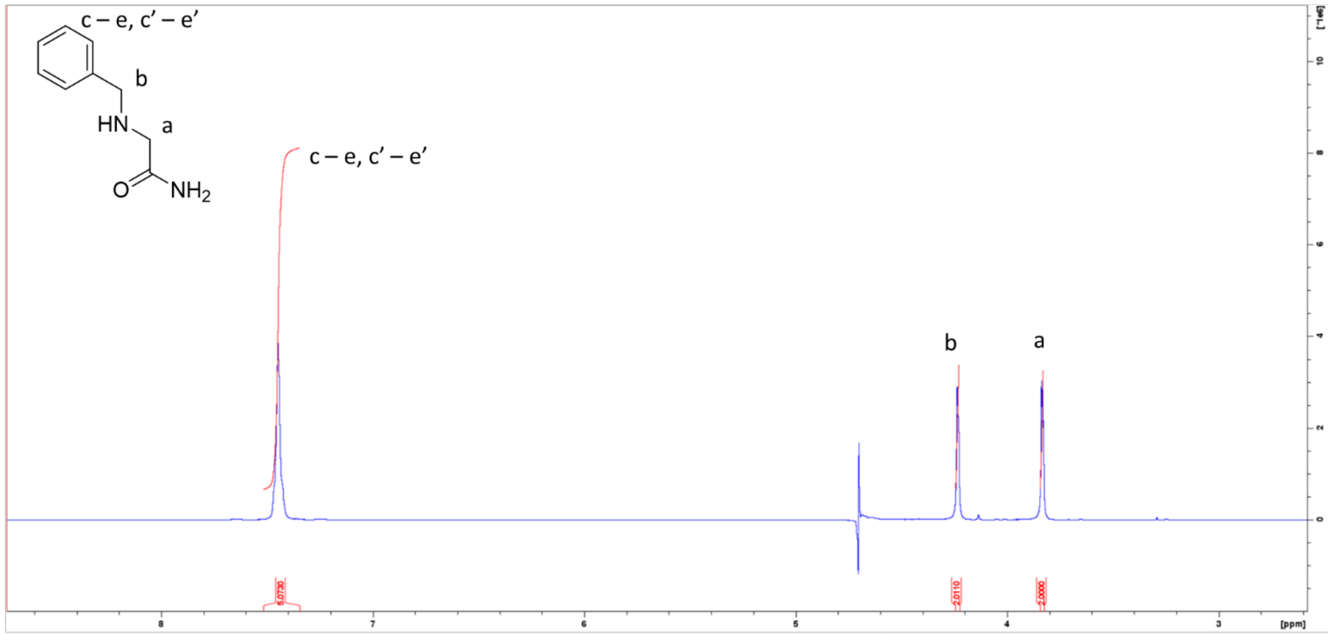

Figure S51. ^{1}H NMR of Nf.HBr (400 MHz, D_2O) δ (ppm): 3.84 (s, 2H), 4.24 (s, 2H) and 7.45 (m, 5H). (Note: peak at 4.70 ppm is H_2O).

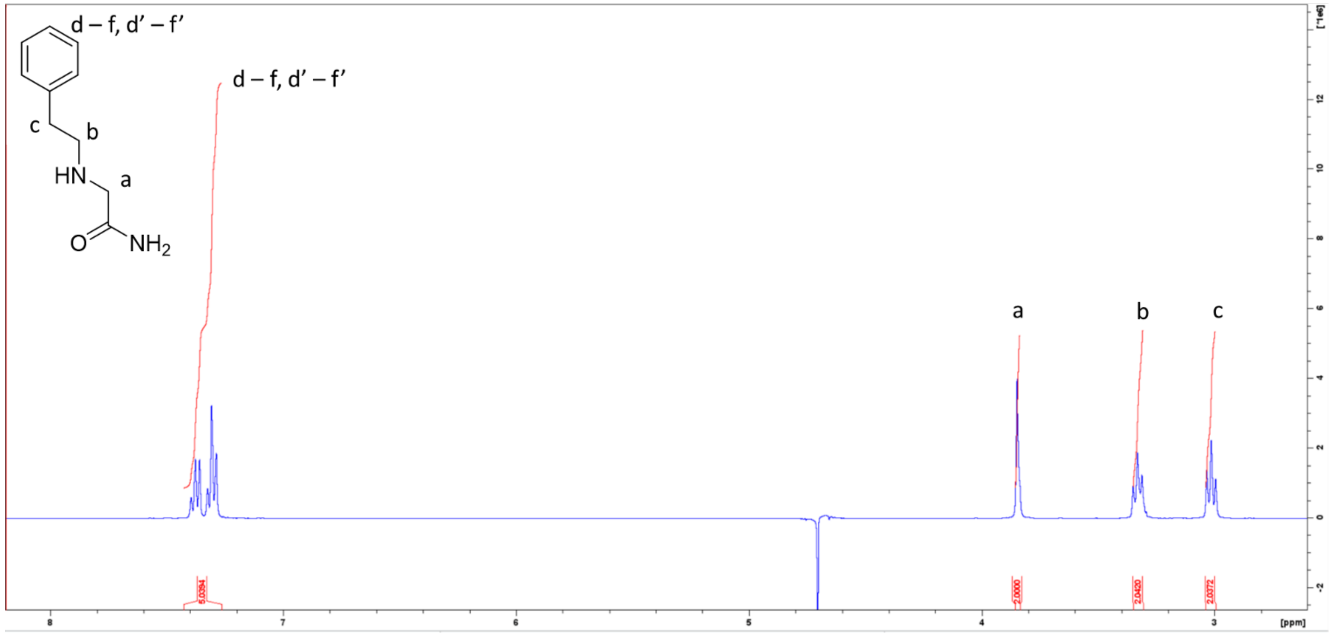

Figure S52. ^{1}H NMR of Nfe.HBr (400 MHz, D_2O) δ (ppm): 3.01 (t, 2H), 3.33 (t, 2H), 3.85 (s, 2H) and 7.34 (dt, 5H). Note: inverted peak at 4.70 ppm is H_2O.

Optical Microscopy

The crystal sample was deposited on glass slides and left to dry. Bright field images were captured using a Nikon Eclipse LV100 and a 10x objective. Polarized light images were captured using a Zeiss Gemini 500 and a 10x objective.

Scanning Electron Microscopy (SEM)

The crystal sample was deposited on an aluminum pin stub and left to dry. Images were captured using FEI Helios NanoLab 660 on samples sputtered with 10 nm of gold.

Atomic Force Microscopy (AFM)

Height image and mechanical property mapping were simultaneously carried out in PeakForce quantitative nanoscale mechanical (QNM) tapping mode using a Bruker Multimode 8 AFM. The optical lever sensitivity (OLS) of the AFM cantilevers (RTESPA-525, Bruker) was calibrated on sapphire surface. The cantilever spring constant was calibrated by Bruker thermal tune. Prior to measuring samples, a flat polystyrene surface of 3.5 GPa Young's modulus was used to confirm the Young's modulus reading of the AFM set up. See further below for AFM step height determination.

Time of Flight Secondary Ionization Mass Spectrometry (TOF-SIMS)

The TOF-SIMS measurements were performed on a Physical Electronics nanoTOF II equipped with 30 KeV Bi^+ liquid metal ion gun (LMIG) as the analysis gun. During the measurements, both low energy (10 eV) Ar^+ gas gun and electron gun were used to neutralize the surface charge. The analysis area is 600 x 600 μm^2. The vacuum was maintained at ~10^{-6} Pa for all measurements.

Molecular Dynamics Simulations

Assembly Simulations

50 monomer molecules were solvated in acetonitrile solvent and neutralized with the anion of interest in a 6 x 6 x 6 nm box. The van der Waals contributions to the potential energy were calculated to a cut-off at 1.2 nm using a force-switch potential modifier at 1.0 nm. Short range electrostatic contributions were calculated below 1.2 nm with Particle Mesh Ewald (PME) summation being used to calculate long range contributions, with a grid spacing of 0.12 nm. Minimisation was done using the steep integrator for 100,000 steps (100,000 steps, emtol = 100 kJ mol^{-1} nm^{-1}). Equilibration (steps = 2,500,000, ts = 2 fs) in the NPT ensemble with a constant temperature of 298.15 K (v-rescale, tau-t = 1.0 ps) and target pressure of 1.0325 bar (Berendsen barostat, tau-p = 1.0 ps, $\beta = 4.5 \times 10^{-5}$ bar^{-1}). The production simulation (steps = 50,000,000, ts = 2 fs) was done using the same target temperature (Nose-Hoover, tau-t = 1.0 ps) and pressure (Parrinello-Rahman, tau-p = 2.0 ps, $\beta = 4.5 \times 10^{-5}$ bar^{-1}). Simulations were performed using Gromacs ver. 2020.7.[4] Parameters for acetic acid, as well as Nab, Nle, Nl were taken directly from the CGenFF parameter server. These were used as they accurately captured both the cation: anion coordination observed by X-ray crystallography (see Figure S35 and Figure 3 in the main text) and the torsional sampling closely reflected the minima in QM calculations. Additional discussion on forcefield selection is provided in Section I.11. To make these Gromacs compatible, a topology file was written using the VMD[5] plugin TopoGromacs.[6] Additional setting for CHARMM simulations within Gromacs details were taken from the Gromacs website.[7] Hydrogen bonds were constrained using the LINCS algorithm.[8]

Interfacial Simulations

80 molecules of either L-NH_2.HCl or V-NH_2.HCl were added to a 4 x 4 x 12 nm box and solvated with acetonitrile. After this the dimensions were then expanded to 4 x 4 x 30 nm to create a vacuum at both sides of the solvent slab. Non-bonded parameters were handled as in the assembly simulations in the absence of phase boundaries. Simulations were then done in the NVT ensemble

for 250 ns to maintain this system structure. Parameters for the amidated amino acids were taken from the Charmm36m forcefield.[16] Sytem preparation involved initial minimisation, followed by a short 100 ps equailibration in the NVT ensemble during which the molecules were restrained in position place to enable solvent and ions to equlibrate. In both these latter stages the velocity rescaling thermostat (V-rescale) was used with tau-t = 0.1 ps to maintain a temperature of 298 K, solvent and ions were coupled seperately to the amidated amino acids.

Quantum Mechanical Calculations

All structures were constructed using Gaussview[1] and calculations were done using Gaussian16.[9] Each peptoid amide was optimized at the MP2/6-31G(d) level of theory and a frequency calculation was also done to ensure that the structure represented an energy minimum (keywords: Opt+Freq). The χ_2 torsion, defined as $N_{ter} – C_\beta – C_\gamma – C_\delta$, was scanned at 12° intervals for 32 steps (keyword: Opt=ModRedundant, total = 384°) and from this the torsion scan between -180° and 180° eas extracted. Energies were converted to kcal mol^{-1} using the constant (1 Hartree = 627.5095 kcal mol^{-1}).

Attenuated Total Reflectance Fourier Transform Infrared Spectroscopy (ATR-FTIR)

Spectra were recorded using an Agilent 5500 Series FT-IR over a range 4000 – 650 cm^{-1} with a resolution of 4 cm^{-1} and 121 scans. Measurements were made in triplicate. After background collection, crystalline material was pressed onto the diamond ATR crystal to entirely cover it.

HPLC Measurements

Each amine was initially dissolved in 600 µL D_2O after NMR analysis (~8 mg/mL). 10 µL of sample was analysed via RP-HPLC using a Nucelosil 100-5 C18 column and a 30 minute 5% to 95% acetonitrile in water gradient with 0.1 % TFA added.

Single Crystal X-Ray Diffraction.

All crystallographic measurements were made with monochromatic Cu radiation (λ = 1.54184 Å) using a Rigaku Synergy-i diffractometer. Raw data processing utilised the program CrysalisPro.[10] The structure was solved using direct methods and was refined against F^2 to convergence using all unique reflections and the program Shelxl,[11] as implemented within WinGX.[12] Multiple samples were examined at a variety of temperatures. All were twinned and many gave poor quality diffraction patterns. The structure presented was treated as a twin by a 180° rotation about [0 1 0] and the reflection data was formatted as a hklf 5 file. Selected crystallographic and refinement parameters are given in Table S3 and Table S4 in the Supplementary Data section (pages 27-28). Full information in cif format has been deposited with the CCDC as reference number 2376002 and 2340476 for Nle.HBr and Nab.HBr respectively.

AFM Step Height Estimation

The line profiles obtained from AFM topography images are shown in Figure S11 and in Figure S26. They exhibit a sawtooth pattern due to an artefact from routine line flattening applied to the topography data. This removed small mismatches in height positions as the AFM tip scanned one line of the surface after another. While the procedure was successful in removing the line-by-line discrepancies and revealed the terrace structure (e.g., Figure S9), the procedure assumed that sample features were well distributed and that the overall surface topography was flat—feature heights were assumed to simply vary an average height position. However, the IFC terrace structure meant that the imaged areas had higher heights at one end of an image than the other, and line flattening removed this overall topography. Hence, the originally flat terraces appeared as

longer upward sloping segments in the line scans and the terrace steps appeared as short slopes dropping in height, resulting in a sawtooth pattern.

To correct this artefact, we applied the procedure shown below. A corrected line profile is shown in Figure 1D in the main text. Note, however, that the average step heights calculated after the corrections were found to be virtually identical to those calculated from the original data (Figure S10). This is unsurprising since the line flatterning procedure is a simple line-by-line height translation and the slope artefact is in any case relatively minor. Nonetheless, we had developed the correction steps below to satisfy ourselves that our step height measurements were robust.

Step 1: In the native NanoScope software, line profiles was drawn on the AFM image obtained after routine first order line flatterning and exported as a text (*txt) file.

Step 2: Due to the terrace steps of our crystalline surfaces, there is a real overall slope in the topography which has been artificially removed by the initial flattening procedure. Thus, the line profiles obtained exhibit artificial "line-drifts" with constant linear gradients (Figure S53). Rather than arbitrarily estimating corrections to recover flat terrace profiles, the gradient was partially corrected with a linear best fit line across both the terrace and step features. This gradient was subtracted from all points of a line profile giving a plot with no artificial increase in height and this was confirmed by re-evaluating the gradient of this new data which in all cases was essentially zero.

Step 3: An simple script was applied to algorithmically calculate all steps in the line profiles: Iterating across the line profile, the change in height (z) was estimated between points i and i + 1. Where this was $\Delta z \geq 0.5$ nm, the algorithm stepped forward to i+2, i+3, etc. When Δz no longer decreased this procedure was stopped and the maximum difference of all Δz values was taken as the step height.

Step 4: This was done for 10 line scans and estimates of the average step height were made before and after the correction in steps 1 and 2 (Figure S10). It was found that there was *no meaningful difference* in the average step height before and after corrections. All traces used are shown in Figure S11 and Figure S26.

Note: the test condition, $\Delta z \geq 0.5$ nm, was validated by trying various step heights and enumerating how many candidate steps ($N_{samples}$) were found for each Δz test. When a plateau was found in the plot of Δz_{test} vs. $N_{samples}$ this was deemed an appropriate test height. For both Nab and Nle this condition was satisfied at $\Delta z_{test} = 0.5$ nm.

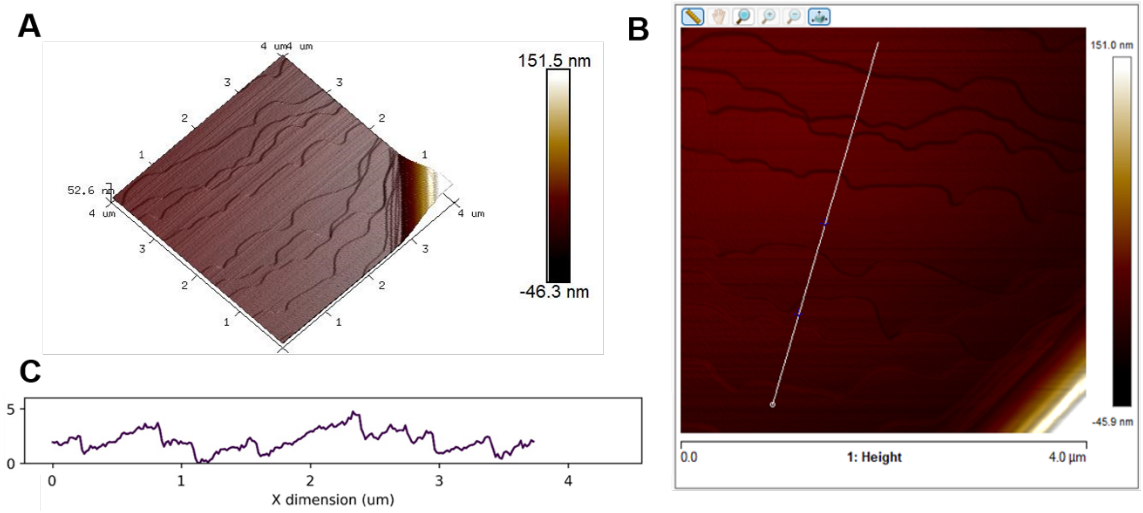

Figure S53. Illustration of the process for step-height estimation via AFM measurement, showing the Nle.HBr data as example. A) Line flatterning was applied to the original AFM data. B) Line profiles were then drawn using the native NanoScope software across the parts of the surface exhibiting terrance steps. Typically 10 profiles were obtained. C) An example of these traces from these step heights were calculated following steps 3 and 4 described in the text.

III. REFERENCES

(1) Dennington, R.; Keith, T. A.; Millam, J. M. GaussView Version 6, 2019.

(2) RDKit: Open-Source Cheminformatics. https://www.rdkit.org.

(3) Wildman, S. A.; Crippen, G. M. Prediction of Physicochemical Parameters by Atomic Contributions. *J. Chem. Inf. Comput. Sci.* **1999**, *39* (5), 868–873. https://doi.org/10.1021/ci990307l.

(4) Abraham, M. J.; Murtola, T.; Schulz, R.; Páll, S.; Smith, J. C.; Hess, B.; Lindahl, E. GROMACS: High Performance Molecular Simulations through Multi-Level Parallelism from Laptops to Supercomputers. *SoftwareX* **2015**, *1–2*, 19–25. https://doi.org/10.1016/j.softx.2015.06.001.

(5) Humphrey, W.; Dalke, A.; Schulten, K. VMD: Visual Molecular Dynamics. *Journal of Molecular Graphics* **1996**, *14* (1), 33–38. https://doi.org/10.1016/0263-7855(96)00018-5.

(6) Vermaas, J. V.; Hardy, D. J.; Stone, J. E.; Tajkhorshid, E.; Kohlmeyer, A. TopoGromacs: Automated Topology Conversion from CHARMM to GROMACS within VMD. *J. Chem. Inf. Model.* **2016**, *56* (6), 1112–1116. https://doi.org/10.1021/acs.jcim.6b00103.

(7) *Force fields in GROMACS*. https://manual.gromacs.org/current/user-guide/force-fields.html.

(8) Hess, B.; Bekker, H.; Berendsen, H. J. C.; Fraaije, J. G. E. M. LINCS: A Linear Constraint Solver for Molecular Simulations. *J. Comput. Chem.* **1997**, *18* (12), 1463–1472. https://doi.org/10.1002/(SICI)1096-987X(199709)18:12<1463::AID-JCC4>3.0.CO;2-H.

(9) Frisch, M. J.; Trucks, G. W.; Schlegel, H. B.; Scuseria, G. E.; Robb, M. A.; Cheeseman, J. R.; Scalmani, G.; Barone, V.; Petersson, G. A.; Nakatsuji, H.; Li, X.; Caricato, M.; Marenich, A. V.; Bloino, J.; Janesko, B. G.; Gomperts, R.; Mennucci, B.; Hratchian, H. P.; Ortiz, J. V.; Izmaylov, A. F.; Sonnenberg, J. L.; Williams-Young, D.; Ding, F.; Lipparini, F.; Egidi, F.; Goings, J.; Peng, B.; Petrone, A.; Henderson, T.; Ranasinghe, D.; Zakrzewski, V. G.; Gao, J.; Rega, N.; Zheng, G.; Liang, W.; Hada, M.; Ehara, M.; Toyota, K.; Fukuda, R.; Hasegawa, J.; Ishida, M.; Nakajima, T.; Honda, Y.; Kitao, O.; Nakai, H.; Vreven, T.; Throssell, K.; Montgomery, J. A., Jr.; Peralta, J. E.; Ogliaro, F.; Bearpark, M. J.; Heyd, J. J.; Brothers, E. N.; Kudin, K. N.; Staroverov, V. N.; Keith, T. A.; Kobayashi, R.; Normand, J.; Raghavachari, K.; Rendell, A. P.; Burant, J. C.; Iyengar, S. S.; Tomasi, J.; Cossi, M.; Millam, J. M.; Klene, M.; Adamo, C.; Cammi, R.; Ochterski, J. W.; Martin, R. L.; Morokuma, K.; Farkas, O.; Foresman, J. B.; Fox, D. J. Gaussian˜16 Revision C.01, 2016.

(10) CrysalisPRO software (2016).

(11) Sheldrick, G. M. Crystal Structure Refinement with *SHELXL*. *Acta Crystallogr C Struct Chem* **2015**, *71* (1), 3–8. https://doi.org/10.1107/S2053229614024218.

(12) Farrugia, L. J. *WinGX* and *ORTEP for Windows* : An Update. *J Appl Crystallogr* **2012**, *45* (4), 849–854. https://doi.org/10.1107/S0021889812029111.

(13) Swanson, H. W. A.; Lau, K. H. A.; Tuttle, T. Minimal Peptoid Dynamics Inform Self-Assembly Propensity. J. Phys. Chem. B 2023, 127 (49), 10601-10614. DOI: 10.1021/acs.jpcb.3c03725.

(14) Tedesco, C.; Schettini, R.; Iuliano, V.; Pierri, G.; Fitch, A. N.; De Riccardis, F.; Izzo, I. Role of Side Chains in the Solid State Assembly of Cyclic Peptoids. Crystal Growth & Design 2019, 19 (1), 125-133. DOI: 10.1021/acs.cgd.8b01137.

(15) Kalita, D.; Sahariah, B.; Pravo Mookerjee, S.; Kanta Sarma, B. Strategies to Control the Cis-Trans Isomerization of Peptoid Amide Bonds. Chemistry – An Asian Journal 2022, 17 (11), e202200149. DOI: https://doi.org/10.1002/asia.202200149.

(16) Huang, J.; Rauscher, S.; Nawrocki, G.; Ran, T.; Feig, M.; de Groot, B. L.; Grubmüller, H.; MacKerell, A. D. CHARMM36m: an improved force field for folded and intrinsically disordered proteins. Nat. Methods 2017, 14 (1), 71-73. DOI: 10.1038/nmeth.4067.
